# Spatiotemporal characterization of glial cell activation in an Alzheimer’s disease model by spatially resolved transcriptomics

**DOI:** 10.1038/s12276-023-01123-9

**Published:** 2023-12-01

**Authors:** Hongyoon Choi, Eun Ji Lee, Jin Seop Shin, Hyun Kim, Sungwoo Bae, Yoori Choi, Dong Soo Lee

**Affiliations:** 1 Department of Nuclear Medicine, Seoul National University College of Medicine, Seoul, Republic of Korea; 2 Department of Nuclear Medicine, Seoul National University Hospital, Seoul, Republic of Korea; 3 Department of Molecular Medicine and Biopharmaceutical Sciences, Graduate School of Convergence Science and Technology, Seoul National University, Seoul, Republic of Korea

**Keywords:** Alzheimer's disease, Translational research, Transcriptomics

## Abstract

The molecular changes that occur with the progression of Alzheimer’s disease (AD) are well known, but an understanding of the spatiotemporal heterogeneity of changes in the brain is lacking. Here, we investigated the spatially resolved transcriptome in a 5XFAD AD model at different ages to understand regional changes at the molecular level. Spatially resolved transcriptomic data were obtained from 5XFAD AD models and age-matched control mice. Differentially expressed genes were identified using spots clustered by anatomical structures. Gene signatures of activation of microglia and astrocytes were calculated and mapped on the spatially resolved transcriptomic data. We identified early alterations in the white matter (WM) of the AD model before the definite accumulation of amyloid plaques in the gray matter (GM). Changes in the early stage of the disease involved primarily glial cell activation in the WM, whereas the changes in the later stage of pathology were prominent in the GM. We confirmed that disease-associated microglia (DAM) and astrocyte (DAA) signatures also showed initial changes in WM and that activation spreads to GM. Trajectory inference using microglial gene sets revealed the subdivision of DAMs with different spatial patterns. Taken together, these results help to understand the spatiotemporal changes associated with reactive glial cells as a major pathophysiological characteristic of AD. The heterogeneous spatial molecular changes apply to identifying diagnostic and therapeutic targets caused by amyloid accumulation in AD.

## Unraveling alzheimer’s: molecular changes across space and time

Researchers used spatially resolved transcriptomic analysis to study spatiotemporal patterns of disease progression in 5XFAD Alzheimer’s disease (AD) mouse models. They found that initial molecular changes related to glial cell activation occurred in white matter (WM) before gray matter (GM) changes. The study also identified distinct activation patterns of microglia and astrocytes that change with AD progression. These findings provide insights into the pathophysiology of AD and could help identify potential molecular targets for AD treatment. This summary was initially drafted using artificial intelligence, then revised and fact-checked by the author.

## Introduction

Advances in single-cell analysis have revealed the diversity of brain cells, and the dynamics of cellular changes in Alzheimer’s disease (AD) have been observed^1–3^. Single-nucleus transcriptome analysis of patients with AD showed major changes in myelination, inflammation, and neuronal survival^2^. In addition, single-cell analysis of AD models revealed molecular changes in microglia and astrocytes. Microglia respond to β-amyloid (Aβ) plaques, and genetic changes in activated response microglia are associated with AD risk genes^4^. Based on these genetic changes in microglia that are specific to AD, disease-associated microglia (DAM) are defined as a microglial subtype^5,6^. The alteration of specific subtypes of astrocytes in AD was also identified and they were defined as disease-associated astrocytes (DAAs)^7^. Despite the identification of the cellular landscape according to AD pathophysiology in specific brain regions, the limitation is the loss of spatial information on cellular networks. Because of the diversity of cellular profiles according to brain regions^8–10^, it is unclear in which brain regions the cellular changes identified in AD are distributed and how they change across brain regions as the disease progresses.

Studies of molecular changes in brain cells as AD pathology progresses should consider regional heterogeneity. According to recent studies, microglia are composed of various subtypes and show high plasticity depending on the surrounding environment^8,11,12^. In the AD model, white matter-associated microglia (WAM), a type of microglia specific only to the white matter (WM), play an important role in the clearance of myelin but do not exist near amyloid plaques^13^. However, the other subtypes of microglia are present and play different roles in gray matter (GM)^13^. In addition, macrophages exist in different subtypes depending on their location, such as the dura mater, subdural meninges, and choroid plexus^14^, and differential patterns of astrocytes and neurons are observed according to the cortical layer^15,16^. These studies show that not only differences in cell subtypes but also functional differences according to brain regions such as GM and WM can be observed. Since brain cells have diversity and dynamics in inter- and intraregions, spatial information within neural circuits is important, and understanding the pathophysiology of AD requires spatiotemporal landscape studies at the molecular level throughout the brain.

Here, we applied spatially resolved transcriptomic analysis in 5XFAD AD models at different ages to identify spatiotemporal patterns of disease progression. First, distinctive brain regions clustered by gene expression were identified, and then molecular changes were analyzed in early (3-month-old) and late (7-month-old) stage AD models. As a result, the analysis confirmed gene patterns that change according to disease progression in each brain region and initial molecular changes related to glial cell activation in WM before the changes in GM. In addition, the spatiotemporal trajectories of the microglia-related gene signature in the spot revealed distinctive activation patterns and found each major marker gene set. These results provide spatiotemporal molecular profiles in the pathophysiology of AD and distinctive activation patterns of microglia and astrocytes that change with AD progression.

## Materials And Methods

### Mice

Three-month- and 7-month-old male 5XFAD mice (Tg6799; on a C57/BL6-SJL background) containing five FAD mutations in human APP (the Swedish mutation, K670N/M671L; the Florida mutation, I716V; and the London mutation, V717I) and PS1 (M146L/L286V) and wild-type mice were used for spatially resolved transcriptomic data and quantitative PCR. Male 5XFAD mice aged 2, 4, 7.5 and 12 months were used for IF imaging of tissue sections. All experimental protocols and animal usage were approved by the Institutional Animal Care and Use Committee at Seoul National University (SNU-181018-6).

### Immunostaining of paraffin-embedded tissue sections

Paraffin-embedded brain tissues were sectioned at 4 μm thickness. Deparaffinization was achieved with xylenes and decreasing concentrations of ethanol. Tissue sections were subjected to antigen retrieval using citrate buffer, pH 6.0, at boiling temperature for 10 min. Following rinsing with TBS, sections were incubated in blocking buffer containing TBS with 0.5% BSA for 1 h at room temperature. Slides were then incubated with primary antibody in blocking buffer overnight at 4 °C. The next day, slides were washed with TBS and then stained with Alexa Fluor secondary antibodies (Thermo Fisher Scientific). Sections were rinsed again and stained with DAPI (1:100; Invitrogen, CA) before being cover-slipped with mounting medium. The primary antibodies used were rabbit Iba1 (1:200; Abcam, Cambridge, UK), rabbit LAMP1 (1:50; Abcam), mouse β-amyloid (6E10) (1:100; BioLegend, London, UK), rabbit β-amyloid (D54D2) XP (1:100; Cell Signaling Technology, CA), mouse GFAP (1:200; Cell Signaling Technology), and rabbit dMBP (1:100; Sigma Aldrich).

### Immunostaining of frozen tissue sections

Frozen brain tissues from 3- and 7-month-old WT and 5XFAD mice were sectioned at 35 μm thickness. The brain sections were incubated in citrate buffer, pH 6.0, at 95 °C for 10 min and then at room temperature for 10 min for antigen retrieval. After washing with PBS, sections were incubated in hydrogen peroxide blocking solution and then in blocking buffer containing PBS with 2% BSA and 0.3% Triton X-100 for 1 h at room temperature. Slides were incubated with primary antibody in blocking buffer overnight at 4 °C. The next day, slides were washed with PBS and then stained with Alexa Fluor secondary antibodies (Thermo Fisher Scientific) or with HRP polymer secondary antibody for DAB reaction (Abcam). After washing with PBS, the sections were stained with DAPI (1:100; Invitrogen) or hematoxylin (Cancer Diagnostics). Mouse MAG (1:100; Sigma Aldrich), mouse CTSS (1:100; Santa Cruz), and rabbit CST7 (1:100; Invitrogen) were used as primary antibodies.

### Thioflavin S staining

The paraffin-embedded sections were deparaffinized in xylene and rehydrated in ethanol solution. The hydrated brain sections were incubated in 1% thioflavin S solution for 5 min and washed with 70% ethanol and distilled water. For the images of stained slides, LEICA confocal microscopy SP8 was used.

### Microdissection of brain tissue and quantitative PCR (qPCR)

Brains were collected and microdissected to obtain samples of the WM and the cortex (free of meninges and choroid plexus). RNA extraction was performed using TRIzol (Thermo Fisher) according to the manufacturers’ protocols. Reverse transcription of RNA was performed using a thermal cycler (Bio-Rad, T100). cDNA samples were diluted and mixed with SYBR green master mix (Takara) before loading as technical triplicates for qPCR on an Applied Biosystems 7500. Primers are specified in Supplementary Table 1.

### Spatially resolved transcriptomic data generation

Prepared brain hemispheres were cryosectioned into thin (10 μm) coronal sections and processed the same day. First, mouse brain sections were sectioned and mounted onto slides on Visium Spatial Tissue Optimization slides (10x Genomics). The tissue permeabilization time was determined by the manufacturer’s protocols (VisiumSpatialTissueOptimization, https://support.10xgenomics.com/spatial-gene-expression/tissue-optimization). Accordingly, tissue was permeabilized for 12 min for Visium Spatial Gene Expression analysis. Before library preparation, tissue sections were methanol-fixed, hematoxylin and eosin (H&E)-stained and imaged on a TissueFAXS PLUS (TissueGenostics, Austria). The slides were merged into a picture of the whole brain using TissueFAXS imaging software. Sections were then permeabilized and processed to obtain cDNA libraries. cDNA libraries were prepared according to the manufacturer’s protocol (VisiumSpatialGeneExpression, https://support.10xgenomics.com/spatial-gene-expression/library-prep). To verify the size of PCR-enriched fragments, we checked the template size distribution by running on an Agilent Technologies 2100 Bioanalyzer. The libraries were sequenced using HiSeqXten (Illumina) with a read length of 28 bp for read 1 (spatial barcode and UMI), 10 bp index read (i7 index), 10 bp index read (i5 index), and 90 bp for read 2 (RNA read).

Raw FASTQ data and H&E images were processed by the Space Ranger v1.1.0 (10X Genomics) pipeline for the gene expression analysis of Visium spatial gene expression library data. Illumina base call files from the Illumina sequencing instrument were converted to FASTQ format for each sample using the ‘mkfastq’ command. Visium spatial expression libraries were analyzed with the ‘count’ command. Image alignment to predefined spots was performed by the fiducial alignment grid of the tissue image to determine the orientation and position of the input image. Sequencing reads were aligned to the mm10 reference genome (mm10-2020-A) using STAR (v2.5.1b) aligner. Gene expression profiling in each spot was performed with unique molecular identifier (UMI) and 10X barcode information.

To evaluate the reproducibility of the spatial transcriptomic data, we conducted a comparative analysis of average gene expression using Pearson correlation. Specifically, we compared the average value of log-normalized counts of all spots between two different samples within each group. This approach allowed us to assess the consistency and reliability of the gene expression measurements in our study, as we obtained two samples for each group.

### Clustering of spatial transcriptomics data

The spots with gene expression data were analyzed with the Seurat package (ver 3.1.2)^17^. Gene counts were normalized using ‘LogNormalize’ methods in Seurat v.3. The top highly variable genes (*n* = 2000) were then identified using the ‘vst’ method in Seurat. The number of RNA counts for each spot and the frequency of mitochondrial gene counts were regressed out in the scaling process. Eight spatial transcriptomic datasets were merged and rescaled. The integration of spatial transcriptomic datasets was performed using the rPCA method in the Seurat package. Principal component analysis was performed using the top highly variable genes. For visualization, dimension reduction was performed using UMAP on the top 30 principal components. Graph-based clustering based on the Louvain community detection algorithm was performed. The resolution was set to 0.2. Markers for each cluster were identified by Wilcoxon rank sum tests for a given cluster vs. other clusters implemented in Seurat as a ‘FindAllMarkers’ function. As spots of spatial transcriptomics data clustered by gene expression are highly associated with neuroanatomical organization, the clusters were designated by anatomical terms^18^. The anatomical location of each cluster was visually identified by comparison with the Allen Mouse Brain Reference Atlas (https://mouse.brain-map.org/static/atlas).

### Differential gene expression analysis on clustering of spots

MAST^19^ in the Seurat function was used to perform differential gene expression analysis. First, we identified differentially expressed genes (DEGs) between two specific clusters (for this study, Cluster 7 vs. Cluster 1, two clusters of thalamus). A gene was considered significant with false discovery rate (FDR)-adjusted *P* < 0.05 and log-fold change (logFC) > 0.3. Volcano plots were drawn by the EnhancedVolcano function in R. In addition, differentially expressed genes were extracted from the comparison of AD and WT mice at 3 months and 7 months. The analysis was also performed using the MAST function after selecting a subset of specific clusters. The cutoff of significantly different genes was FDR-adjusted *p* < 0.05 and log FC > 0.25. Gene Ontology (GO) and KEGG (Kyoto Encyclopedia of Genes and Genomes) pathway analyses were performed with clusterProfiler^20^, which supports statistical analysis and visualization of functional profiles for genes and gene clusters.

### Microglial and astrocyte signature scores

Gene sets of microglia and DAM were selected for the matrices of spatial transcriptomic data to calculate the signature score. The score was calculated with the AddModuleScore function with default parameters in Seurat. DAMscore was visualized by the SpatialFeaturePlot function for identifying spatial distribution patterns. In addition, gene sets of DAAs were selected to calculate the signature score. DAA scores were also visualized by the SpatialFeaturePlot function.

### Cell type enrichment score for spatial transcriptomic data

We utilized CellDART to analyze the cell type scores for the spatial transcriptomic data^21^, considering that the data consisted of spots containing a mixture of various cell types. To estimate the cell type scores, we employed single nucleus RNA-seq data from the mouse cortex and hippocampus^22^. The taxonomy of cell types was established based on a previous publication from the Allen Brain Map (https://portal.brain-map.org/)^22^. For cell type mapping in CellDART, we used 10 marker genes for each cell type and generated pseudospot mixtures by combining 5 cells per pseudospot. These parameters were selected to ensure accurate cell type assignment and reliable representation of the spatial transcriptomic data.

### Spatial coregistration of the immunofluorescence image

The immunofluorescence image of an amyloid plaque (6E10; mouse) obtained from a 7-month-old 5XFAD model was spatially coregistered with the H&E image obtained before spatial transcriptomic data acquisition, i.e., Visium library generation. Notably, the immunofluorescence image of the amyloid plaque was the adjacent slide of the H&E image for spatial transcriptomic data generation. The immunofluorescence image was resized to have a similar size as the H&E images. Cropping and rotation were performed to approximately overlap both images. This process was performed by the scikit-image package (version 0.15.0). For image registration, both images were preprocessed by (1) changing to grayscale, (2) masking with a pixelwise threshold to include the mouse brain, and (3) smoothing using a Gaussian filter (sigma value of 5). The transform function for the coregistration was estimated using the Dipy package (version 1.0.0). The image was linearly transformed by rigid and affine transformation. For the final coregistration, nonlinear warping was performed using SymmetricDiffeomorphicRegistration based on the Symmetric Normalization (SyN) algorithm^23^. After the estimation of transformation function, the immunofluorescence image was transformed for further analysis integrating image patterns and spatial transcriptomic data.

### Integrative analysis of the immunofluorescence image and spatial transcriptomic data

Molecular features associated with tissue image patterns were extracted by the spatial gene expression patterns by deep learning of tissue images (SPADE) tool^24^. The coregistered amyloid plaque immunofluorescence image was used for the input of SPADE. The CNN-derived image features (VGG-16 model) were extracted by 32 × 32 sized patches centered at the spots. For dimension reduction, principal components of image features were used. In this study, the first principal component represented the accumulation of amyloid plaques; thus, genes correlated with the first principal component of image patterns were identified. The top 100 genes according to the regression coefficient (logRC) were selected. GO analysis was performed with clusterProfiler.

### Spatiotemporal trajectory using pseudotime analysis

A gene set with microglial signatures was selected to generate a matrix for spatially resolved gene expression data: *Hexb, Cst3, Cx3cr1, Ctsd, Csf1r, Ctss, Sparc, Tmsb4x, P2ry12, C1qa, C1qb, Tmem119, Tyrobp, Ctsb, Apoe, B2m, Fth1, Lyz2, Trem2, Axl, Cst7, Ctsl, Lpl, Cd9, Csf1, Ccl6, Itgax*, and *Timp2*^5,6,25^. Normalization and PCA were performed using the ‘preprocess_cds’ function from Monocle version 3. The trajectory graph based on the microglial signature gene set was learned by the ‘learn_graph’ function from Monocle. The UMAP plots according to the trajectory were drawn with colors of the clusters using all genes analyzed with Seurat as well as mice. Subsequently, the spots were semiautomatically ordered according to the progression of microglia. The trajectory was automatically learned; however, the direction of order was determined by UMAP plot with mice (AD vs. WT). As the spots of 7-month-old AD mice were located at a specific portion, the center of the UMAP, the spots enriched in 7-month-old AD mice were regarded as ‘late-phase’ pseudotime.

Spatial mapping of pseudotime for each trajectory was performed by selecting the subset of spots included in the selected trajectory. Colors with pseudotime were mapped on the spots of specific locations and mapped using SpatialFeaturePlot from Seurat.

### Statistics

Differentially expressed genes were identified by the aforementioned methods. All *P* values reported in this study were two-tailed and adjusted by false discovery rate. The statistical method incorporated in the specific software was used with a default parameter unless otherwise indicated. Plots in R were created either with the ggplot2 R package or Seurat modified by custom codes for data visualization.

## Results

### Distinctive gene expression patterns commonly found in the GM of AD by spatial transcriptome-based cluster analysis

**Fig. 1:**
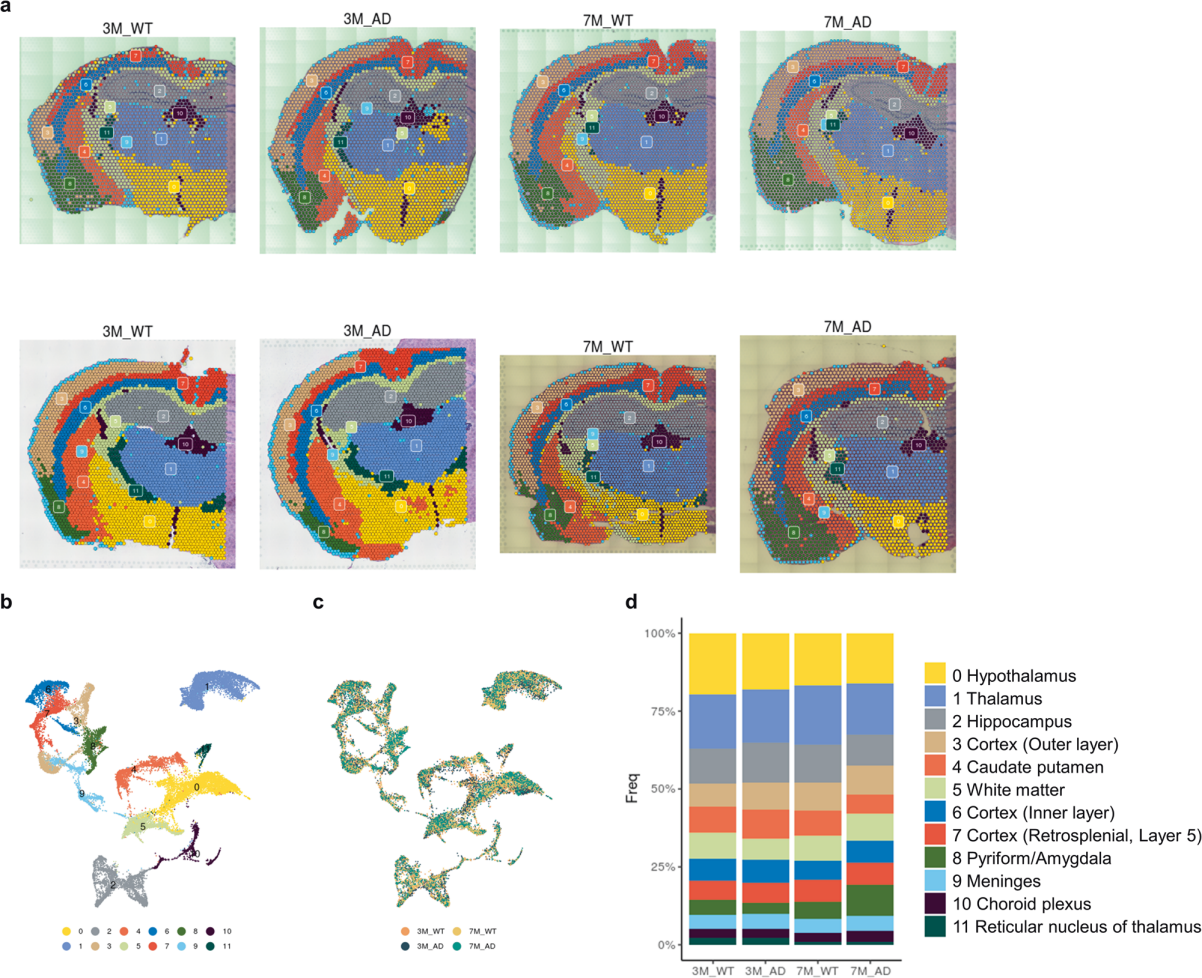
Clusters of spots of spatially resolved transcriptomes in WT and 5XFAD mice. **a** Twelve clusters were identified according to transcriptomic data of 3- and 7-month-old WT and 5XFAD mice. **b** A two-dimensional reduction map, UMAP, colored with clusters is depicted. Each dot represents the transcriptomic data of each spot. **c** A UMAP colored with mouse types is depicted. **d** The frequency of spots of each cluster for different mice is represented. (WT: wild type; AD: Alzheimer’s disease; 3 M: 3-month-old; 7 M: 7-month-old).

Spatial transcriptomic data of the 5XFAD AD model and age-matched wild-type (WT) mice were obtained at 3 and 7 months of age (*n* = 2 for each of 3- and 7-month-old AD and WT mice). To assess the reproducibility of the spatial transcriptomic data within each group, we calculated the average gene expression across all spots. This measure was obtained by analyzing the log-normalized counts. To provide a visual representation of the results, we generated a scatter plot, which is included as Supplementary Fig. 1. Notably, a 7.5-month-old 5XFAD model showed amyloid plaques in the GM, while the 2-month-old AD model did not. The 4-month-old 5XFAD model showed a small number of amyloid plaques in the thalamus and cortex (Supplementary Fig. 2a). In particular, as a result of confirming the amyloid deposition in the brain tissues of the 5XFAD model, which were the same as those for the spatial transcriptomic data, a small proportion of amyloid deposition was observed in the 3-month-old 5XFAD model, and amyloid plaques were also observed in the 7-month-old 5XFAD model (Supplementary Fig. 2b). A total of 26,324 spots containing 32,285 mRNA expression data points were clustered according to the expression patterns only. Accordingly, 12 different clusters were identified. These clusters corresponded to anatomical structures (Fig. 1a). For example, Cluster 3 represented the cerebral cortex, including mainly outer layers, and Cluster 6 represented the inner layers of the cerebral cortex. Cluster 2 included the hippocampus, and Cluster 4 represented the striatum (Supplementary Table 2). The expression data of spots were represented by UMAP plots, a dimension reduction method for visualization^26^ (Fig. 1b). Spots with different mice are also depicted (Fig. 1c). Markers of each cluster were extracted and visualized with a heatmap, and the GO of each cluster was analyzed (Supplementary Fig. 2c, Supplementary Table 2). In addition, we have provided the cell type scores for each cluster in Supplementary Fig. 2d. The cell types were defined based on the classification from the Allen Brain Map (https://portal.brain-map.org/). These scores offer insights into the relative abundance of different cell types within each cluster, enhancing our understanding of the cellular composition in the analyzed data. The frequency of each cluster was represented to identify a specific cluster enriched in the 5XFAD models (Fig. 1d). Of note, to obtain differentially expressed genes for each brain region, clustering of spots was performed after the integration of 8 samples. Thus, clusters represented each brain region.

**Fig. 2:**
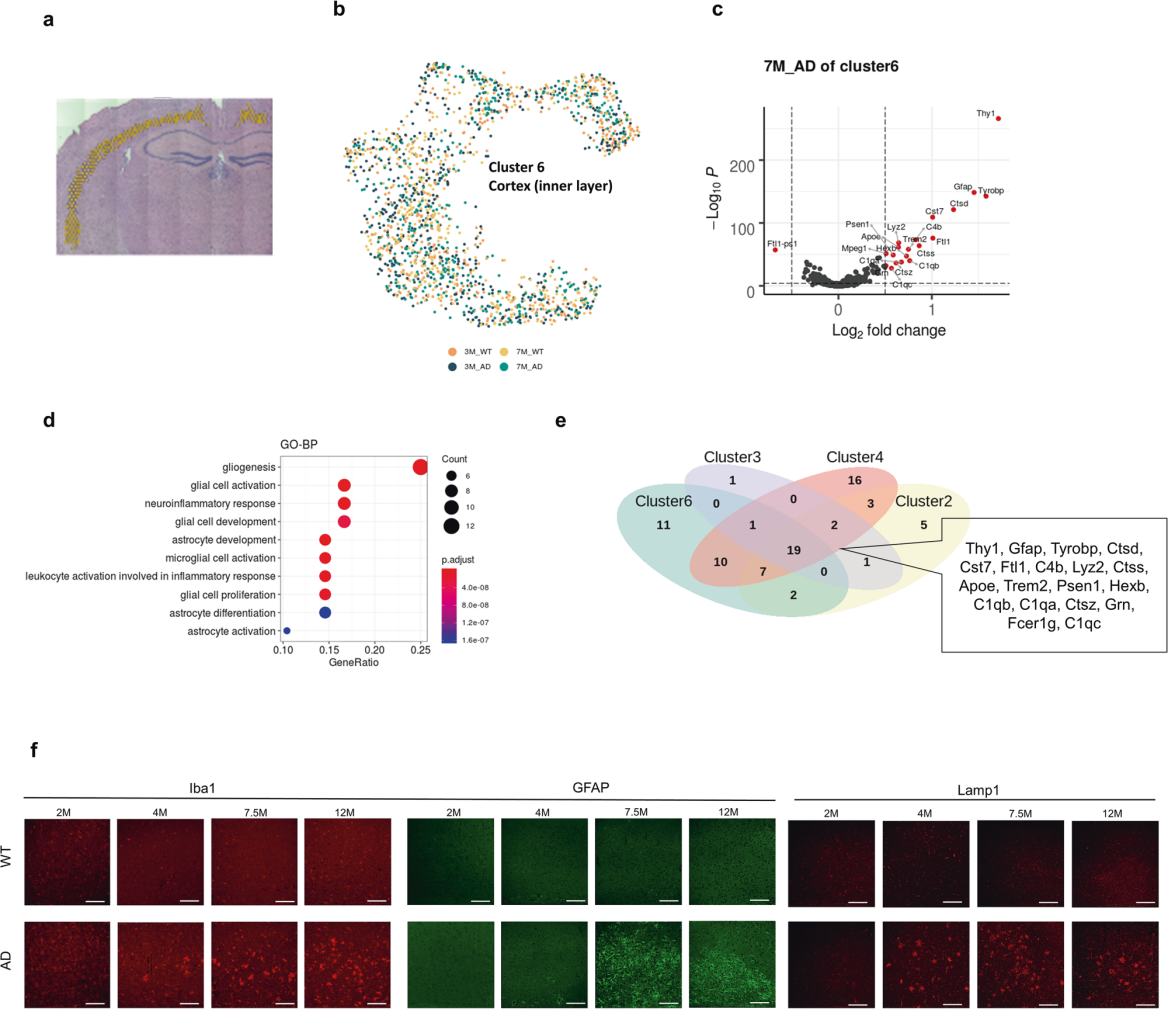
Genes differentially expressed in the gray matter of the 5XFAD model. **a** As a cluster of the cerebral cortex, Cluster 6 (cerebral cortex, inner layer) was selected to find differentially expressed genes in 5XFAD compared with WT mice. **b** UMAP represented spots of Cluster 6 according to the origin of samples. **c** A volcano plot depicting differentially expressed genes of Cluster 6 in the 5XFAD model compared with the WT at 7 months. **d** The upregulated genes in the 7-month-old 5XFAD model were similar to the markers of Cluster 7, which occupied the thalamus of the 7-month-old 5XFAD model. **e** A Venn diagram representing the upregulated genes in the 7-month-old 5XFAD model for different clusters (Clusters 2, 3, 4 and 6). There were 19 commonly upregulated genes in all these GM clusters. **f** Immunostaining of microglia/macrophages (Iba1, red), astrocytes (GFAP, green), and markers of lysosomal function (Lamp1, red) were observed in the cortex at the indicated ages (*n* = 3). Scale bars, 50 μm.

We then analyzed DEGs in specific brain regions of the AD brain. First, genes differentially expressed in the cerebral cortex of AD, Cluster 6, were identified (Fig. 2a). UMAP showed spots according to the origin of the samples (Fig. 2b). In Cluster 6, differential gene expression was found mainly in the 5XFAD model at 7 months, while mice at 3 months showed few differentially expressed genes (Fig. 2c, Supplementary Fig. 3a). The genes highly expressed in the 7-month-old 5XFAD model included *Gfap, Tyrobp, Ctsd, Cst7, Ftl1*, and *C4b*, which are related to inflammation mediated by microglia and astrocytes. GO pathway analysis revealed that upregulated genes in the cerebral cortex of the AD model at 7 months were associated with gliogenesis and neuroinflammation related to microglia and astrocytes (Fig. 2d). In addition, lysosome and apoptosis activity was enriched in the cerebral cortex of the 7-month-old AD model (Supplementary Fig. 3a). We additionally tested DEGs in the AD brain according to the regions. Accordingly, the hippocampus (Cluster 2), striatum (Cluster 4), and outer cortex (Cluster 3) shared genes differentially expressed at 7 months (Fig. 2e, Supplementary Fig. 3b). The commonly upregulated genes in these GM regions of the 7-month-old AD model are represented in Fig. 2e. Indeed, IF images showed increased reactivity of microglia, astrocytes, and lysosomal function in the cerebral cortex using antibodies against Iba1, GFAP, and LAMP1 from the AD model older than 4 months (Fig. 2f). Cell type scores of astrocytes and microglia were determined using CellDART. Notably, despite variations across clusters, no consistent rise was noted in microglia and astrocytes in 7-month-old AD models (Supplementary Fig. 3c).

### Distinctive spatial pattern of DEGs within WM in early AD

**Fig. 3:**
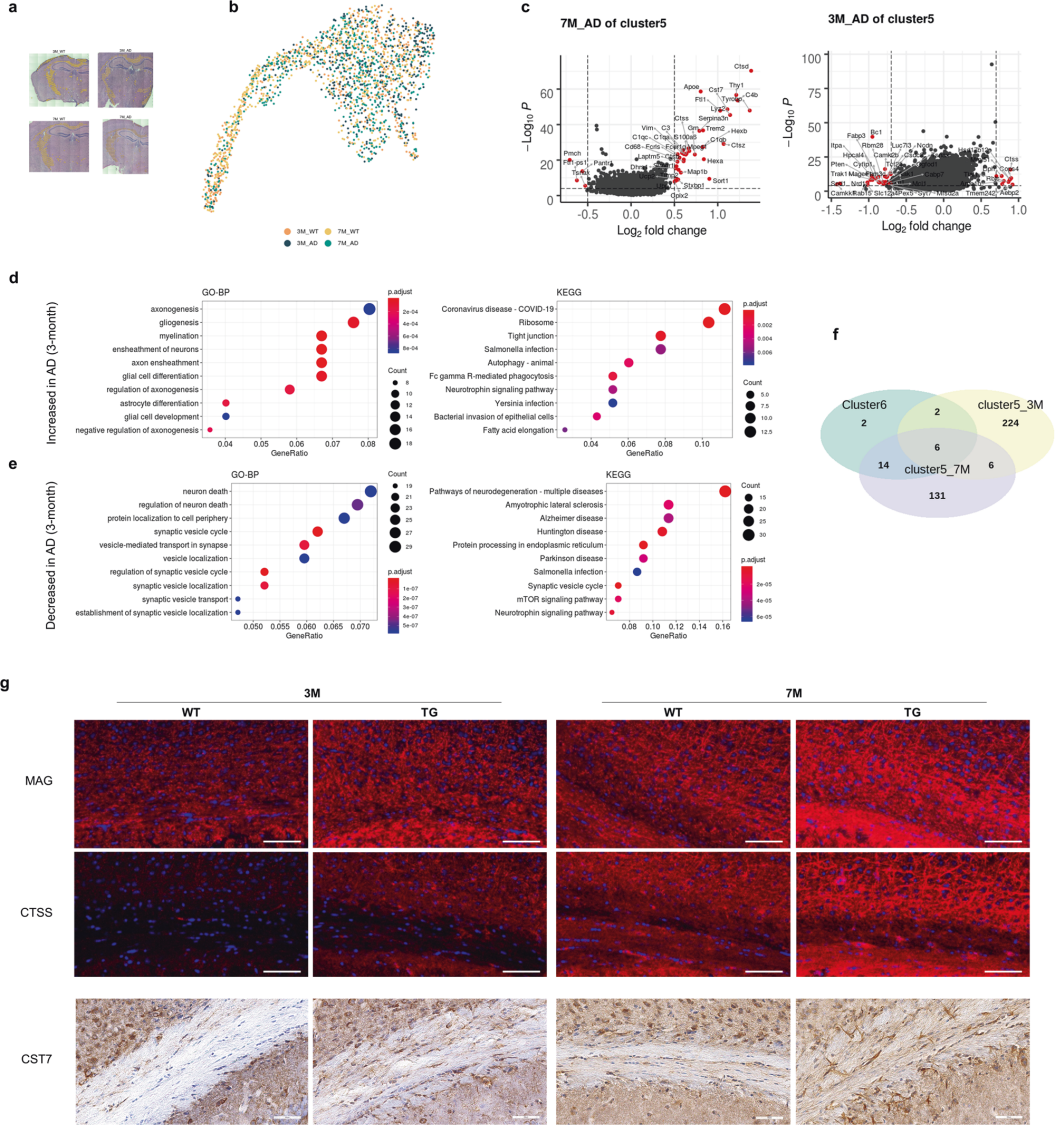
Genes differentially expressed in the WM of the 5XFAD model as an early change. **a** Cluster 5 represents WM regardless of mouse type. **b** UMAP represents transcriptomic profiles of Cluster 5 according to mouse type. **c** Many differentially expressed genes were identified in the 5XFAD model at 3 months and at 7 months. **d** As an early change in WM (i.e., 3-month-old 5XFAD), upregulated genes were related to axonogenesis, gliogenesis, and myelination. **e** Downregulated genes in the 3-month-old 5XFAD model were related to neuronal death and the synaptic vesicle cycle. **f** The upregulated genes of Cluster 5 in 3-month-old 5XFAD mice were different from the upregulated genes of GM in 7-month-old 5XFAD mice. However, more shared upregulated genes of Cluster 5 in 7-month-old 5XFAD mice with those of GM were identified than those of Cluster 5 in the 3-month-old AD model. **g** Immunostaining of MAG (red), CTSS (red), and CST7 (brown) was observed in the corpus callosum at the indicated ages (*n* = 3). Scale bars, 50 μm.

Cluster 5, which included mainly WM, showed distinctive patterns in the transcriptome (Fig. 3a). A UMAP represented spots of the clusters in WM (Fig. 3b). In Cluster 5, striking DEGs were identified in 3-month-old mice as well as 7-month-old mice (Fig. 3c). At 3 months, the 5XFAD model showed that many genes were upregulated compared with the WT. These genes included *Tmem242, Cops4, Ctss, Nucb1, Mrpl51, Hsd17b12, Tial1, Smad4*, and *Arpc1b*. In addition, many genes were downregulated compared with the WT. These genes included *Camkk1, Snd1, Metap1d, Dnal1, Rab15, Slc12a4, Nrd1*, and *Itpa*. The upregulated genes in the WM of 3-month-old AD brains were associated with axonogenesis, gliogenesis, and ensheathment of neurons (Fig. 3d). qPCR analysis also showed that the mRNAs encoding *Mag* and *MOG* were significantly increased in the 3-month-old 5XFAD model compared to the control and 7-month-old 5XFAD models, respectively (Supplementary Fig. 4a). The decreased genes in the WM of the 3-month-old 5XFAD model were associated with neuron death and synaptic vesicles (Fig. 3e). Common genes differentially expressed in the WM of 3-month- and 7-month-old 5XFAD model mice and those in the GM are depicted in Fig. 3f. Early changes in molecular markers were also found in the immunostaining study. Immunohistochemistry results of fluorescence images with MAG and CTSS and chromogenic images of CST7 showed increased expression in the WM in the 3-month-old 5XFAD model (Fig. 3g). In addition, the lysosomal function shown by staining with anti-LAMP1 also showed the same pattern. Reactive microglia were gradually increased in the WM, while they were increased in the 7.5-month-old 5XFAD model, and a few fluorescent spots were identified in the 4-month-old 5XFAD model (Supplementary Fig. 4b). At 7 months, many DEGs were also identified in Cluster 5. Increased expression was found of *Ctsd, Tyrobp, C4b, Lyz2, Cst7*, and *Ctsz*, which were similar to upregulated genes in other clusters of GM, such as the cerebral cortex, hippocampus and striatum. qPCR analysis revealed significant increases in *Cst3, Trem2*, and *Tyrobp* in both WM and GM at 7 months in the 5XFAD model, and the upregulated expression of *Apoe* was identified only in WM (Supplementary Fig. 4a). At 7 months, enriched GO terms included myeloid cell activation and gliogenesis (Supplementary Fig. 4c). Accordingly, genes differentially expressed in the WM of the 3-month-old 5XFAD model were distinctive from others, particularly in the 7-month-old 5XFAD model identified in GM (Cluster 6). However, genes differentially expressed in the WM of the 7-month-old 5XFAD model included the more common genes differentially expressed in the GM (Fig. 3f). The DEGs of each cluster are summarized in Supplementary Table 3.

### Integrative analysis with the immunofluorescence image of Aβ

**Fig. 4:**
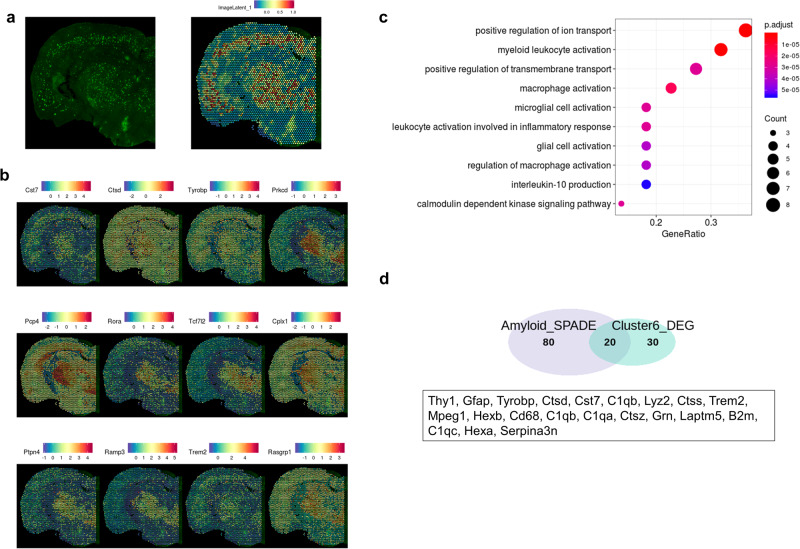
SPADE analysis showed genes spatially associated with amyloid plaque images. **a** Antiamyloid plaque IF image in a 7-month-old 5XFAD model used for sequencing was spatially registered to the histology image of the spatially resolved transcriptome. The latent image (‘ImageLatent1’) derived by the first principal component of deep learning-based features is represented (right). **b** Top 12 genes spatially associated with ‘ImageLatent1’, analyzed by SPADE analysis. **c** GO terms of the spatially associated genes are represented. **d** Among genes spatially associated with ‘ImageLatent1’, 20 genes were upregulated in Cluster 6 of the 7-month-old 5XFAD model.

As the 7-month-old 5XFAD model showed amyloid plaques on the IF image, we integrated this image with spatial transcriptomic data. The image was spatially registered with the H&E image of the AD brain at 7 months using nonlinear transformation (Supplementary Fig. 5a). Therefore, we obtained registered images of amyloid plaques that corresponded to spots of spatial transcriptomic data using adjacent slides of the same brain tissue used for sequencing. Molecular features spatially correlated with the amyloid plaque image patterns were estimated using the SPADE tool^24^. Briefly, the image patch corresponding to the spot was extracted to estimate image features derived by a convolutional neural network model, and then correlated genes were identified. The first principal component (‘ImageLatent_1’) of the CNN-derived image features was mapped (Fig. 4a). The spatial distribution of ‘ImageLatent_1’ corresponded visually to the pattern of amyloid deposits, while other principal components were different from the degree of amyloid deposits (Supplementary Fig. 5b). The top 12 genes spatially correlated with ‘ImageLatent_1’ are represented (Fig. 4b). According to GO analysis using the top 100 correlated genes with ‘ImageLatent_1’, these genes represented the regulation of ion transport and myeloid leukocyte activation (Fig. 4c). Genes associated with amyloid plaque image patterns (SPADE genes) partly overlapped with DEGs at 7 months in the cerebral cortex (Fig. 4d). These genes, *Thy1, Gfap, Tyrobp, Ctsd, Cst7, C1qb, Lyz2, Ctss, Trem2, Mpeg1, Hexb, Cd68, C1qb, C1qa, Ctsz, Grn, Laptm5, B2m, C1qc, Hexa*, and *Serpina3n*, were increased in the 7-month-old 5XFAD model in the cortex and associated with amyloid plaque image patterns.

### Spatial distribution of DAM and DAA signatures

**Fig. 5:**
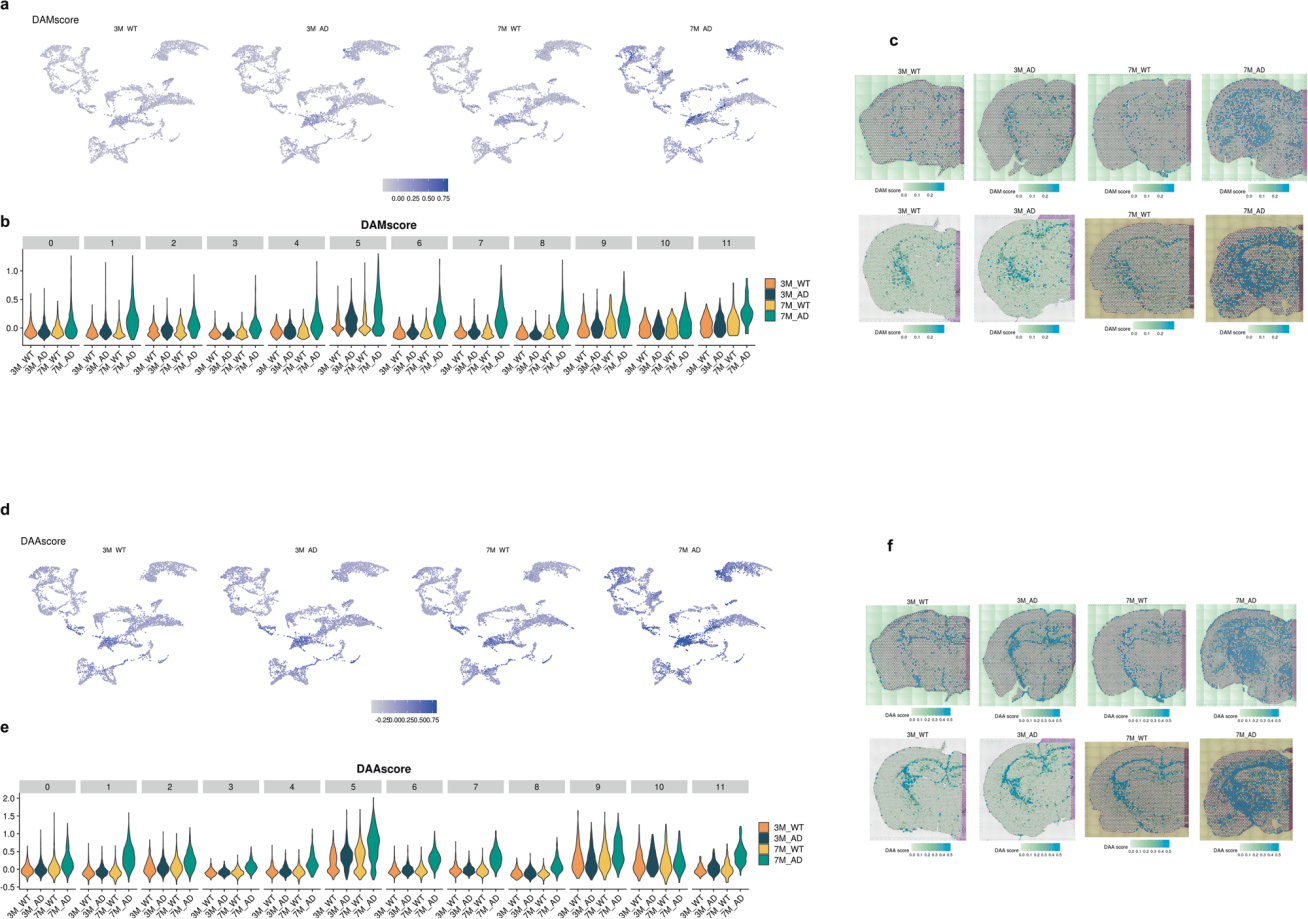
Spatial distribution of DAM and DAA signatures. **a** The DAM score is represented by UMAP plots. **b** DAM scores of each cluster were compared and overall increased in the 7-month-old 5XFAD model. The DAM score was also increased in the 3-month-old 5XFAD model in Cluster 5 (WM). **c** The spatial distribution of the DAM score showed an increase in the GM of 7-month-old 5XFAD mice and a relatively prominent increase in the WM of 3-month-old 5XFAD mice. **d** The DAA score was represented by UMAP plots. **e** DAA scores of each cluster were compared and overall increased in 7-month-old 5XFAD mice. The DAA score was also increased in the 3-month-old 5XFAD model in Cluster 5 (WM). **f** The spatial distribution of DAA scores showed an increase in the 7-month-old 5XFAD model, particularly in the WM and thalamus regions, and a slight increase in the WM region in the 3-month-old 5XFAD model.

Spatial transcriptomic data revealed that AD-related transcriptomic changes at 3 months involved mainly the WM, and then the features of reactive glial cells commonly extended to the cortex, striatum and hippocampus. Thus, we analyzed the disease-associated activation signatures of microglia and astrocytes, major players in AD-related neuroinflammation, in terms of spatial and temporal patterns. Spatial patterns of activated microglia, represented by the DAM signature, were identified. A module score of the DAM signature (DAMscore) was calculated by using the expression values of *Lpl, Cst7, Axl, Itgax, Spp1, Cd9, Ccl6*, and *Csf1* for each spot^5,6^ (Supplementary Fig. 6a). The qPCR results showed significantly increased *Axl, Itgax*, and *Cst7* levels in both WM and GM in the 7-month-old 5XFAD model (Supplementary Fig. 6b). IF images with anti-Iba1 revealed that microglia in the internal capsule and corpus callosum were increased at 4 months in the 5XFAD model, and they were clearly increased in the cortex and thalamus at 7.5 months (Supplementary Fig. 6c). DAM scores of spots were represented by UMAP plots, which revealed spots with increased DAM scores in the 7-month-old 5XFAD model (Fig. 5a). DAM scores of clusters were compared according to the mouse types (Fig. 5b). In addition, the spatial distribution of DAM scores was represented (Fig. 5c). Notably, these results showed that the DAM score was relatively high in the WM of the 3-month-old 5XFAD model (i.e., Cluster 5). A statistical comparison of DAM scores between 3-month-old 5XFAD and WT mice for each cluster is presented in Supplementary Fig. 6d. In addition, WT mice also showed relatively high DAM scores in border regions such as the meninges (Cluster 9), choroid plexus (Cluster 10), and reticular nucleus of thalamus (Cluster 11) (Fig. 5c, Supplementary Fig. 6e), which suggested the heterogeneity of myeloid cells in the brain, identified as border-associated macrophages (BAMs) that substantially share gene sets of DAM^14^. Despite the increased DAM score in the WM of the 3-month-old 5XFAD model, the expression of *Mbp*, a marker of oligodendrocytes, was not changed in the 3-month-old or 7-month-old 5XFAD model (Supplementary Fig. 6f).

We also analyzed the age-dependent spatial pattern of astrocytes activated only in AD, as indicated by DAA signatures. The module score of the DAA signature (DAA score) was expressed in patterns of *Ggta1*, *Gsn*, *Osmr*, *Vim*, *Serpina3n*, *Ctsb*, and *Gfap*^7^. The DAA score increased at 3 months in the WM (i.e., Cluster 5), and further increases were identified at 7 months in areas such as the thalamus and cerebral cortex (Fig. 5d, e, Supplementary Fig. 6g). Increased reactivity of astrocytes was observed, especially in the cortical cortex and thalamus at 7 months (Fig. 5f). qPCR analysis revealed that *Gfap* and *Serpina3n* were significantly upregulated in both WM and GM in the 7-month-old 5XFAD model, while *Vim* showed a tendency toward increased expression levels only in WM (Supplementary Fig. 6g). IF images of GFAP showed increased expression levels in the internal capsule and corpus callosum of a 4-month-old 5XFAD model. At 7 months, in addition to WM, *Gfap* expression was increased in the thalamus and cortex (Supplementary Fig. 6h). Thus, similar changes in spatial patterns were identified for the pathological conditions of DAM and DAA.

### Spatiotemporal reactive microglial patterns identified by trajectory analysis

**Fig. 6:**
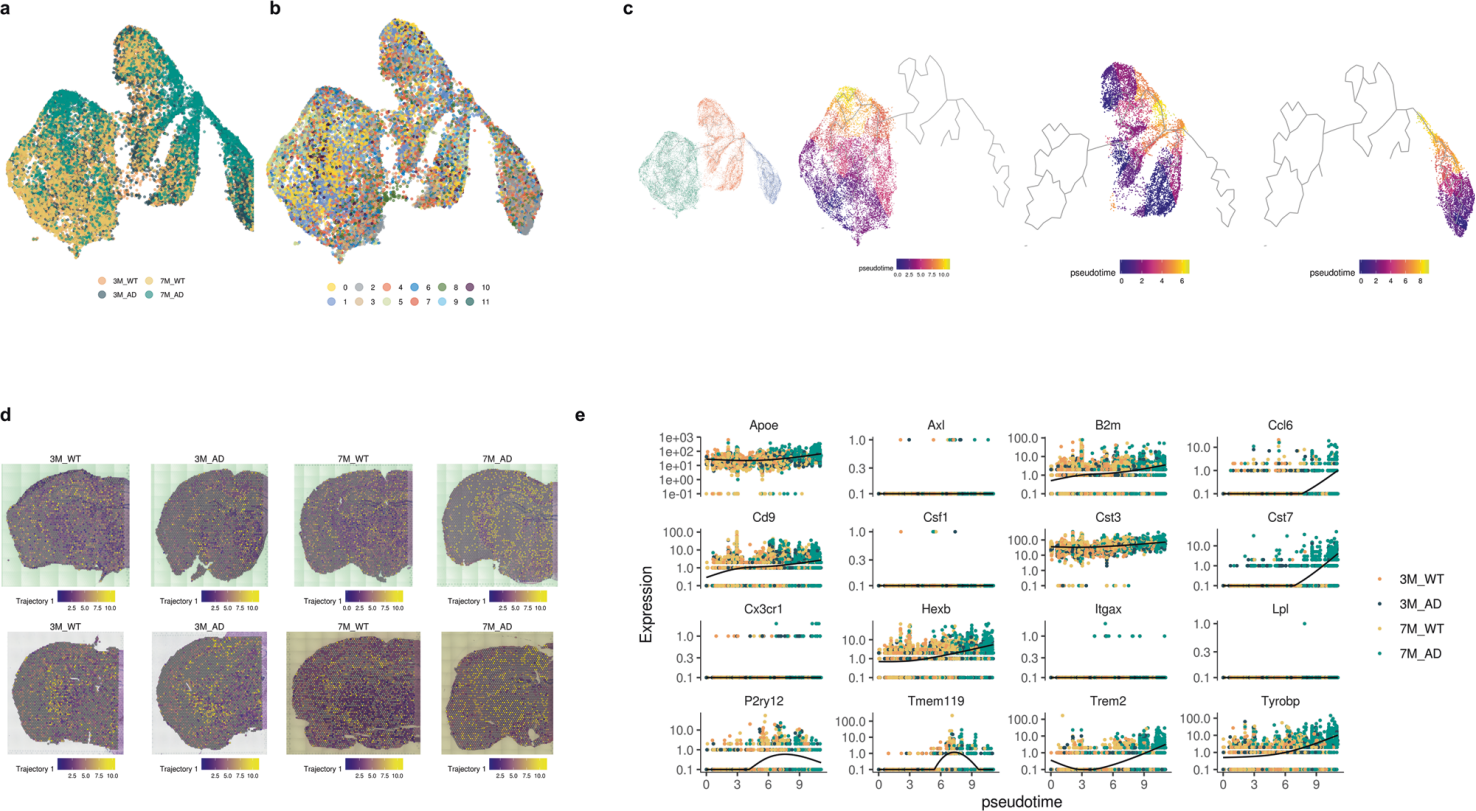
Trajectory inference of microglial signatures of spots. **a** UMAP represents the microglial signature-based distribution. Notably, clusters determined by the transcriptome were mixed. **b** UMAP of microglial signatures colored according to mouse type is presented. **c** Trajectory inference analyzed with Monocle3 showed three different trajectories of spots of microglial signatures. **d** The first trajectory is represented with spatial maps and colored by pseudotime. Notably, the direction of pseudotime was determined by the predominant spots of the 7-month-old 5XFAD model in the center portion of UMAP. **e** The expression of microglial signature genes according to the pseudotime of trajectory 1 is presented. *Trem2, Ccl6*, and *Cst7* were increased at the late phase of pseudotime, while *Axl, Lpl*, and *Csf1* were negative until the late phase of pseudotime.

Genes related to reactive microglia and homeostatic microglia were selected to find spatiotemporal patterns of microglial signatures in the brain (the gene sets are summarized in Supplementary Table 4)^5,6^. Using these gene sets, the trajectory of spots was inferred using Monocle 3^27^. The UMAP plot based on the microglial gene sets showed that spots of 7-month-old 5XFAD mice showed a trend of clustering in the center and left-upper portion of the UMAP plot compared with the spots of other brains (Fig. 6a). In addition, UMAP depicted a heterogeneous distribution in terms of the clusters representing anatomical regions (Fig. 6b). Accordingly, the direction of the trajectory of microglial activation according to disease progression could be inferred. The expression of key genes in microglia is presented in UMAP plots (Supplementary Fig. 7a). These plots revealed that the expression of activated microglial genes, including *Trem2*, *Cst7*, and *Ccl6*, was increased according to the trajectory. According to the distribution of activated microglial genes and brain samples, three different trajectories were defined based on trajectory analysis (Fig. 6c).

The activated status of trajectory 1, increased pseudotime, was found in both WT and 5XFAD models despite different spatial distributions (Fig. 6d). The activated status of trajectory 1 was widely identified in most clusters of 7-month-old 5XFAD mice. However, 3-month-old 5XFAD brains showed an activated status of trajectory 1 in WM (Fig. 6d. Supplementary Fig. 7b). These results corresponded to previous results of early changes in WM. Notably, spots with an activated status of trajectory 1 were found in the meningeal area (Cluster 9) in WT mice (Supplementary Fig. 7b). In other words, trajectory 1 microglial activation was found in WT as well as 5XFAD mice, although 5XFAD mice showed spatially different patterns: the activated status of trajectory 1 in the entire brain at the late stage (7 months). Gene expression according to the activation pattern of trajectory 1 is represented (Fig. 6e). The activated status of trajectory 1 showed high *Ccl6, Cst7, Trem2*, and *Tyrobp* and low expression of *Axl, Csf1*, and *Lpl*.

**Fig. 7:**
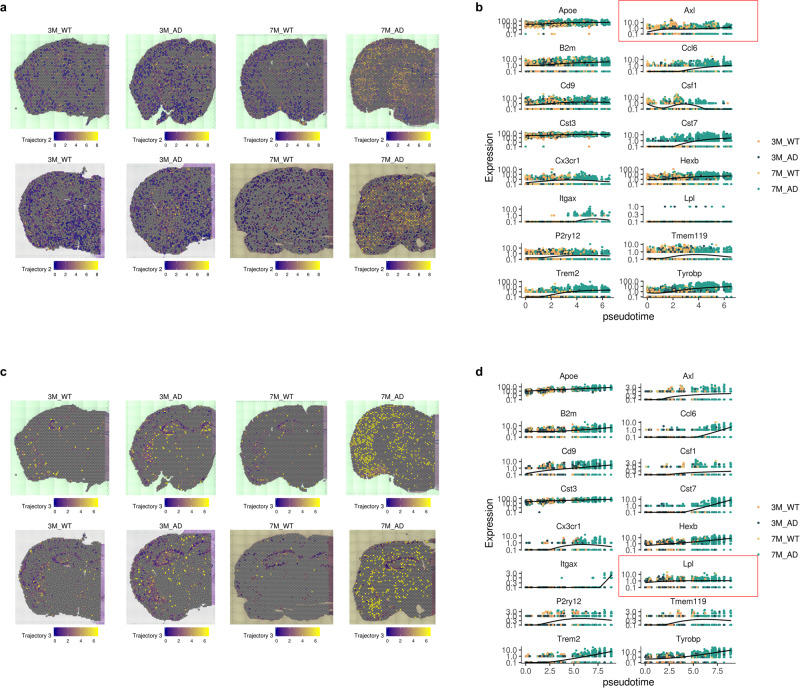
Trajectories of microglial signatures specific for AD. **a** Another trajectory, trajectory 2, was spatially mapped. The late phase of trajectory 2 was found only in the 7-month-old 5XFAD model, particularly in thalamo-cortical regions. **b** The expression of microglial signature genes according to the pseudotime of trajectory 2 is represented. *Axl* was highly expressed in trajectory 2 regardless of pseudotime. **c** Trajectory 3 was spatially mapped. This trajectory commonly included spots of the hippocampus. The late pseudotime of trajectory 3 was found only in the 7-month-old 5XFAD model. **d** The expression of genes according to the pseudotime of trajectory 3. Notably, *Lpl* was highly expressed in trajectory 3 regardless of pseudotime.

The other activated status of trajectory 2 was found in the AD model, while the WT showed early pseudotime status of trajectory 2 (Fig. 7a). The spatial distribution of trajectory 2 was also different from the spatial distribution of trajectory 1. In the WT and 3-month-old 5XFAD models, spots with early pseudotime status were diffusely distributed in the thalamus and cerebral cortex (Fig. 7a). The distribution was not changed in the thalamo-cortical regions of the 7-month-old 5XFAD model. However, spots on the thalamus and cerebral cortex showed late pseudotime status of trajectory 2. Trajectory 2 showed high *Axl* expression regardless of the pseudotime, which was different from trajectory 1. Other markers, such as increased *Trem2* according to pseudotime, were similar to the markers of trajectory 1 (Fig. 7b).

The activated status of trajectory 3 was also associated with AD, while WT and 3-month-old mice showed early status on trajectory 3 (Fig. 7c). The spatial distribution was different from other trajectories. The spots included in trajectory 3 were identified in the hippocampus and amygdala. The activated status of trajectory 3 was found in the cortical cortex and thalamus of a 7-month-old 5XFAD model. In addition, spatial spots related to trajectory 3 pseudotime were identified in the striatum of the early AD model at 3 months (Fig. 7c). Trajectory 3 was characterized by high expression of *Lpl* regardless of the pseudotime, which was different from other trajectories (Fig. 7d). Of note, the spatial distribution of *Lpl* was increased in the hippocampus of WT mice according to in situ hybridization (ISH) data from the Allen Brain Atlas as well as spatial transcriptomic data (Supplementary Fig. 7c).

## Discussion

We observed molecular features of the GM of 5XFAD models in the age-matched WT. The GM, including the cerebral cortex and hippocampus, showed a similar pattern of molecular changes. At 3 months, few molecular changes were observed, and there was a marked increase in reactive glial cell-related changes at 7 months. The dramatic activation of glial cells in the GM, an area where severe amyloid deposition occurs, demonstrated a close relationship with AD progression. Therefore, we concentrated on genes spatially associated with amyloid deposit patterns and investigated them by integrative analysis of the spatially resolved transcriptome and IF imaging. The top DEGs extracted by SPADE included genes related to glial activation, such as *Cst7* and *Ctsd*, while a few genes, such as *Prkcd, Rora*, and *Tcf712*, were not significantly upregulated in the 5XFAD model. These genes were associated with the regulation of ion transport. The spatial association suggested the possibility of functional interaction between amyloid deposits and membrane potential. Amyloid aggregation on the cell membrane results in lipid bilayer disruption and cell leakage, which are potential mechanisms of toxicity^28,29^. Of note, *Prkcd* was recently identified as a cerebrospinal fluid biomarker related to neurodegeneration induced by beta-amyloid^30^. In addition, we identified 20 genes that were correlated with amyloid deposit patterns and upregulated in GM at 7 months in 5XFAD mice. Among these genes, *Serpina3n* was different from the others that were involved mainly in microglial activation. Our results show that the expression level of *Serpina3n* gradually increased as AD pathology progressed dramatically in WM and GM. *Serpina3n* is commonly detected in astrocytes and activated oligodendrocytes and is linked to increased amyloid accumulation, even though the mechanism is not clear^31^. According to a recent study, *Serpina3n* secreted by activated oligodendrocytes plays a role in promoting amyloid plaque deposition^32^. Furthermore, *Serpina3n* is a key marker of recently identified DAAs and is spatially adjacent to amyloid plaques^7^. These results confirmed that upregulated genes in AD spatially associated with amyloid plaques were consistent with previously identified markers functionally related to Aβ. Therefore, we suggest that changes in glial cells around amyloid plaques and changes in glial cells throughout the whole brain occur simultaneously.

An interesting biological finding in the current study is the initial changes in WM in 5XFAD models before distinct changes in GM. The major alteration of DEGs in the GM was confirmed in 7-month-old 5XFAD mice, which had pronounced amyloidosis. Of note, genes that were not significantly altered in GM but differently altered in WM were identified at 3 months. Global molecular changes in terms of cell types were also observed in human data of late-stage AD, while cell-specific molecular changes were found in the brain associated with early AD pathology^2^. In this regard, the early change before the upregulation of DEGs in global GM could provide evidence of the role of WM changes independent of amyloid deposits in the GM. This finding also partly corresponds to a recent study with single nucleus RNA-seq analysis in 5XFAD mice, which identified a reactive oligodendrocyte population^32^. The study showed oligodendrocyte changes in 5XFAD mice, even in Trem2^−/−^ 5XFAD mice, even though they suggested that oligodendrocytes were affected by amyloid deposits. In addition, a previous study with a spatial transcriptome in APP^NL-G-F^ mice suggested early alterations in a gene coexpression network related to myelin^33^. Although the early alteration of oligodendroglial cells is a common finding with our work, the previous paper showed downregulation in the highest amyloid accumulation area. However, myelination process genes were generally increased in App^NL-G-F^ mice^33^, particularly in WM regions. The generally upregulated genes related to oligodendrocytes were consistent with our findings. Our results suggested that WM changes functionally related to axonogenesis and myelination were, at least partly independent of amyloid plaques considering their early changes. According to the human single nucleus RNA-seq data of Alzheimer’s disease obtained from the prefrontal cortex, alterations in myelination-related genes were also observed, while they were changed across various cell types as well as oligodendrocytes in early AD pathology^2^. Furthermore, the WM of a 7-month-old 5XFAD model showed upregulation of genes related to reactive glial cells, as found in GM, such as *Trem2, Cst7*, and *Apoe* (Fig. 3c). Thus, the results implied sequential molecular changes in WM: early changes independent of amyloid plaques followed by Trem2-dependent inflammatory signatures at the late phase.

From the analysis to identify DEGs in each cluster, 5XFAD mice were compared to age-matched WT mice using spatial transcriptomics at the spot level. However, to ensure the robustness of our analysis accounting for individual biological variation and avoid potential biases from randomly shuffled data, we tried pseudobulk RNA-seq by summing gene counts from each spot within a cluster and compared the groups. In addition, we shuffled the group labels of the samples to determine whether DEGs were differently extracted despite the small number of each group (*n* = 2) (Supplementary Fig. 8). The results showed more DEGs from pseudobulk data in the brain region compared with the shuffled group, indicating that the DEGs identified in our study were hardly due to individual variation. Furthermore, the key changes were validated by qRT‒PCR and immunofluorescence in different mice, as shown in Supplementary Fig. 4 and Fig. 2f. Nonetheless, in future work, more samples integrated with spatial as well as bulk RNA-seq data could provide more robust information.

In addition, AD-specific DAM and DAA signature analysis characterized the spatial distribution. Although the division of reactive glial cells into fixed categories is still controversial^34^, we used well-described activation markers to distinguish reactive glial cells from homeostatic glial cells^5^. Our study showed that the signatures were increased in WM in the 3-month-old 5XFAD model. As pathology progressed, genes in disease-associated glial cells were upregulated in GM with more pronounced expression levels than in WM (Fig. 5). This result is consistent with the finding of confirming the initially altered DEGs of WM in the 3-month 5XFAD model. Although the signatures are different from those of DAM, the increased DAM signature in the WM at 3 months suggested Trem2-dependent microglial activation regardless of changes in GM. A recent report characterized WAMs engaged in clearing myelin with single-cell RNA sequencing^13^. In an AD mouse model, WAMs displayed partial activation of the DAM gene signatures, allowing the identification of various aspects of DAM activity. WAMs, but not DAMs, appeared in 3-month-old APP^NL-G-F^ mice, which could reveal that myelin degeneration starts earlier than amyloid pathology^13^. Similar to microglia, DAAs showed the same activation patterns, initially from the WM to the thalamus and cortex area. In summary, the WM-to-GM transition provides important information about the pathological changes in AD progression, but further studies are needed to determine whether this transition occurs dependently and how glial cells communicate in each region. Even though it was difficult to distinguish changes in specific cells or an increase in cells with specific markers in the current spatial transcriptomic data, our focus on the changes in gene expression or related cell types in glial cell activation-related signatures allowed us to project how these changes are represented at the whole brain level.

Spatiotemporal changes in microglial signatures as key players in neuroinflammation were analyzed using a trajectory model. In this analysis, we selected spots containing genes related to DAM as well as homeostatic microglia and analyzed genes from all cells in the spot, including microglial signatures. Therefore, it is expected that not only changes in microglia but also the characteristics of the cells that change together were analyzed. As a result, the trajectory was divided into three distinct patterns (Fig. 6c). The late phase of the distinctive trajectories reached a similar point associated with DAM genes. Trajectory 1 included both WT and 5XFAD mice despite the different phase distributions of each group. However, trajectories 2 and 3 were found exclusively in the 7-month-old 5XFAD model. Trajectory 2 was associated with positive *Axl* expression, and trajectory 3 was associated with positive *Lpl* expression (Fig. 7). Notably, *Lpl* expression was normally found in the hippocampus as an early phase of trajectory 3 (Supplementary Fig. 7c). Although spatiotemporal patterns could not directly reflect microglia themselves, they suggested spatially distinctive activation patterns of glial cells. Furthermore, these results suggested that microglial activation patterns in AD could be region-specific and not a single molecular pathway. Even with the same DAM-related genes, there were genes that existed exclusively in AD pathology and were even present in the WT. Our results suggest that the subdural area in AD and even the white matter area in the WT have activated microglial signatures but were limited to trajectory 1 (Supplementary Fig. 7b). These areas showed that *Axl* and *Lpl* were negative, indicating that they differed from the AD-specific activated status. With a similar result, *Axl* expression was relatively high in only the 7-month-old 5XFAD model compared to *Itgax* and *Cst7*, which showed a significant increase even at 3 months (Supplementary Fig. 7b), indicating that *Axl* may be expressed at the late stage of AD pathology. Moreover, work done by Chen et al. highlighted spatial changes in the activation-related genes of glial cells throughout the brain, employing spatial transcriptomics to achieve this^33^. Previous work also suggested a widespread change in glial cell gene activation patterns across the brain, reinforcing the idea that these changes are a key aspect of the disease’s pathology. These similarities between our work and that of a previous report underscore the potential significance of these genes and pathways in the context of AD^33^. In addition, the amyloid plaque-induced genes (PIGs) identified by Chen et al. largely coincided with our list of amyloid-associated genes and differentially expressed genes (DEGs) in 7-month-old 5XFAD mice. Out of 20 genes, 16 were also included in the PIGs list: *Tyrobp, Ctsd, Cst7, C1qb, Lyz2, Ctss, Trem2, Mpeg1, Hexb, C1qb, C1qa, Ctsz, Laptm5, B2m, C1qc*, and *Serpina3n*. Our analysis using spatial transcriptomics with relatively higher resolution could show spatiotemporal patterns of DAM signatures, which were not the same across the whole brain. Based on our study, microglia expressing the DAM signature genes exhibited a variety of activation patterns, suggesting that these distinct microglia could be present in specific regions of the brain. This can provide valuable information regarding the impact of AD pathology on different brain areas. These findings shed light on which microglial changes are more pronounced in certain brain regions and can help identify potential molecular targets for AD treatment.

Overall, spatially resolved transcriptomic data from WT and AD mouse models of different ages showed spatiotemporally heterogeneous patterns of AD pathology. Despite our efforts to validate our findings using multiple approaches, one limitation of our study is the lack of direct translation into human AD from the mouse model. Detailed comparison of single-cell and/or spatial transcriptomic data of AD in humans and mice is needed. Therefore, future studies could benefit from a more comprehensive validation regarding spatiotemporal heterogeneity associated with AD pathology, particularly in human AD. Moreover, our study acknowledges the possible influence of specific mouse models and experimental methodologies. The integrative approach of AD models solely for tau as well as amyloid plaques from different experimental methods and mouse models introduces inherent complexities, which could potentially lead to model-specific or method-dependent biases. To definitively eliminate these potential biases, a comparative analysis involving the use of identical methods across various AD models would be necessary, a task that extends beyond the scope of our current investigation. In addition, it was difficult to distinguish changes in specific cells or global cell types in the spatial transcriptomic data analyzed. While our approach allowed us to project how these changes are represented at the whole-brain level, future studies could benefit from more refined methods, such as spatial transcriptomics with higher resolution, to identify changes in specific cell types or markers in spatial transcriptomic data. Nonetheless, as cellular changes in a small specific region of the brain could not represent changes in the whole brain, our results provide resources for AD-related transcriptomic changes in gross-scale coverage with high-resolution spatial resolution.

### Supplementary information

Supplementary Figures and Tables

Supplementary Table 3

## Data Availability

The raw and processed spatially resolved transcriptomic sequencing data are available at the Gene Expression Omnibus database under the accession code GSE174321. Other data are available upon request.
