# Supplementary material for: Spatiotemporal characterization of glial cell activation in an Alzheimer’s disease model by spatially resolved transcriptomics
Source: Exp Mol Med. 2023 Dec 1;55(12):2564–75. doi: 10.1038/s12276-023-01123-9 (PMC10767047; doi:10.1038/s12276-023-01123-9)
Supplement: Supplementary file 1 — Supplementary Figures and Tables [file 12276_2023_1123_MOESM1_ESM.pdf]

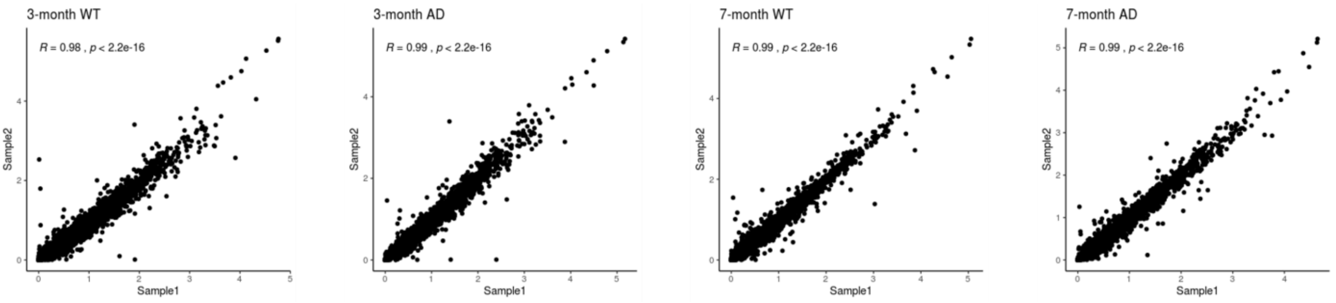

Supplementary Fig. 1. Reproducibility of pairs of spatial transcriptomics data. Reproducibility of pairs of spatial transcriptomics data. The dataset used in this study consists of samples from the AD model of 5XFAD mice and age-matched wild-type (WT) mice at 3 and 7 months of age. For each group (3-month-old AD, 3-month-old WT, 7-month-old AD, and 7-month-old WT), two samples were analyzed. To assess the consistency of the spatial transcriptomic data within each group, the average gene expression across all spots was calculated. Each spot represents an individual gene, and the log-transformed averaged normalized counts across all spots are depicted on both the x-axis and y-axis. The scatter plot visually represents the results of this reproducibility evaluation, allowing for an interpretation of the gene expression patterns within each group and an understanding of the reproducibility levels observed.

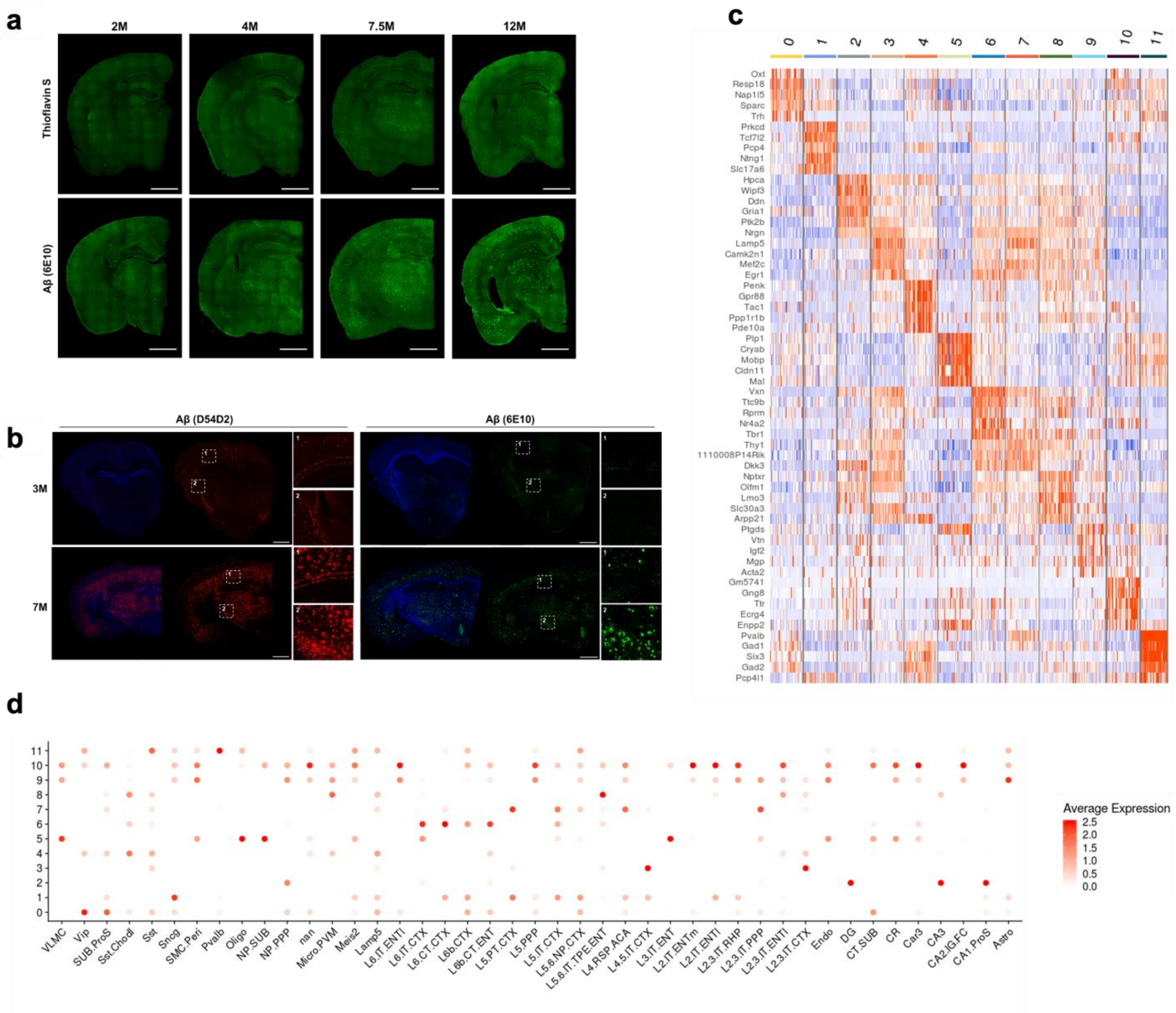

Supplementary Fig. 2. Age-dependent accumulation of β-amyloid in AD model brains and marker genes of spots of spatial transcriptome.

(a) Immunofluorescence imaging on whole brain tissue slides from 2, 4, 7.5 and 12-months-old AD model is shown (n=3). Coronal brain sections were stained with Thioflavin S (*above*) and 6E10 antibody showing Aβ accumulation (*below*). (b) Immunofluorescence imaging for the brain tissues, 3-and 7-month-old 5XFAD, is shown. These images were acquired from the same brain from which spatially resolved transcriptome data were acquired. Aβ was stained with D54D2 antibody (red) and 6E10 antibody (green), and Aβ accumulation in the GM of the 3-month-old AD model was observed. In the inset, 1 indicates corpus callosum and cortex, 2 indicates internal capsule and thalamus. The images were acquired using tile-scaling LEICA confocal imaging software. Scale bars, 1 mm. (2M: 2-month-old; 4M: 4-month-old; 7.5M: 7.5-month-old; 12M: 12-month-old). (c) A heatmap of markers of each cluster was represented. Top 5 marker genes of the clusters were selected. (d) Cell type scores for each cluster was represented. Cell type scores were estimated according to the transcriptomics-based cell type taxonomy of Allen atlas.

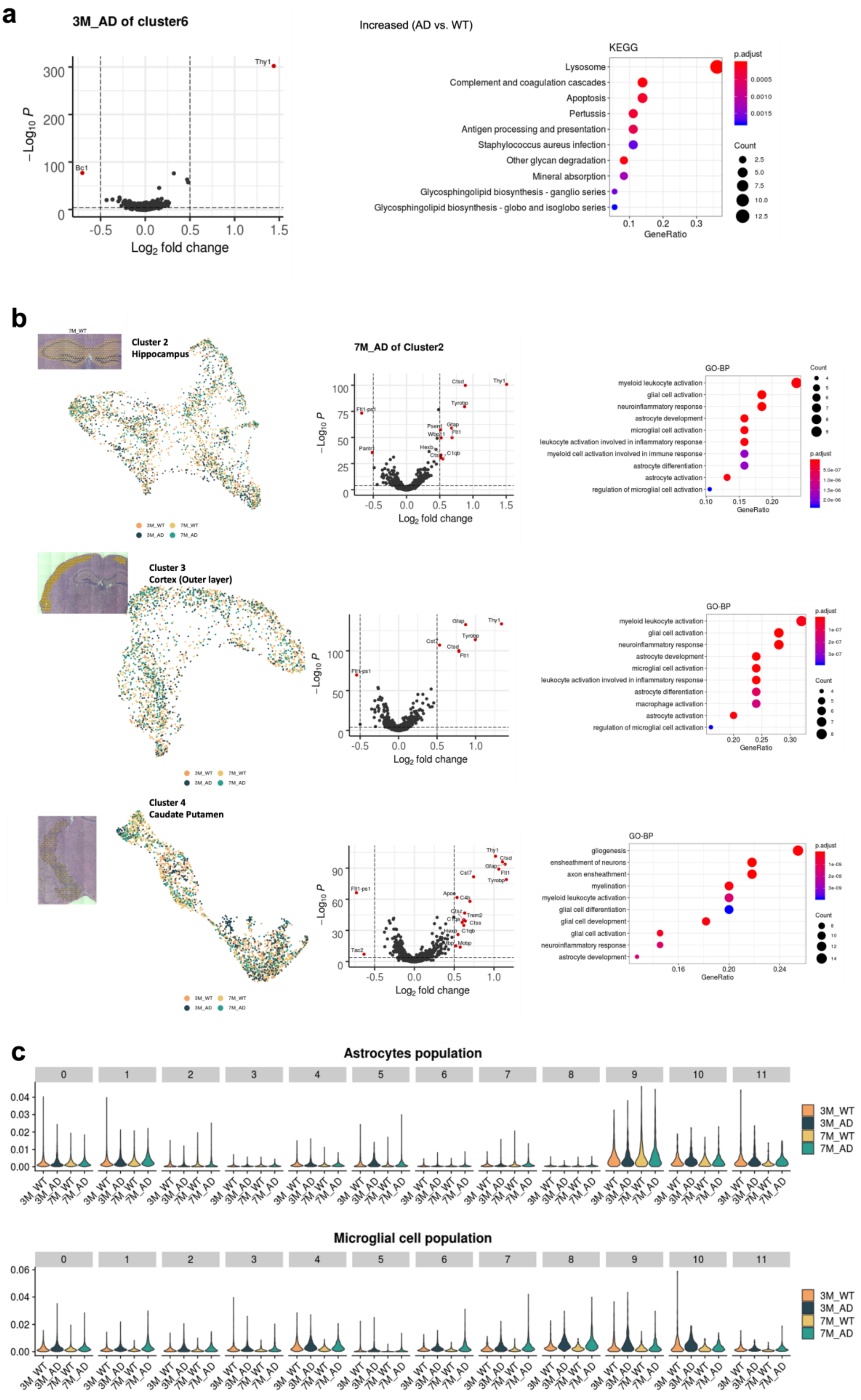

Supplementary Fig. 3. Differentially expressed genes and GO of the AD model according to clusters. (continue)

(a) Differentially expressed genes in the cerebral cortex of AD model, cluster 6. Differentially expressed genes in 3-month-old AD model compared with WT (*left*). KEGG pathways of differentially expressed genes in 7-month-old AD model (*right*). (b) Differentially expressed genes and GO terms of upregulated genes in 7-month-old AD model compared with WT. The analyses were performed in cluster 2 (hippocampus), cluster 3 (outer layer of cerebral cortex), and cluster 4 (caudate/putamen). (c) The cell type scores of astrocytes and microglia were estimated using CellDART. Even though the population of these cell types varied across clusters, no consistent increase was observed in these cell types particularly in 7-month-old AD models.

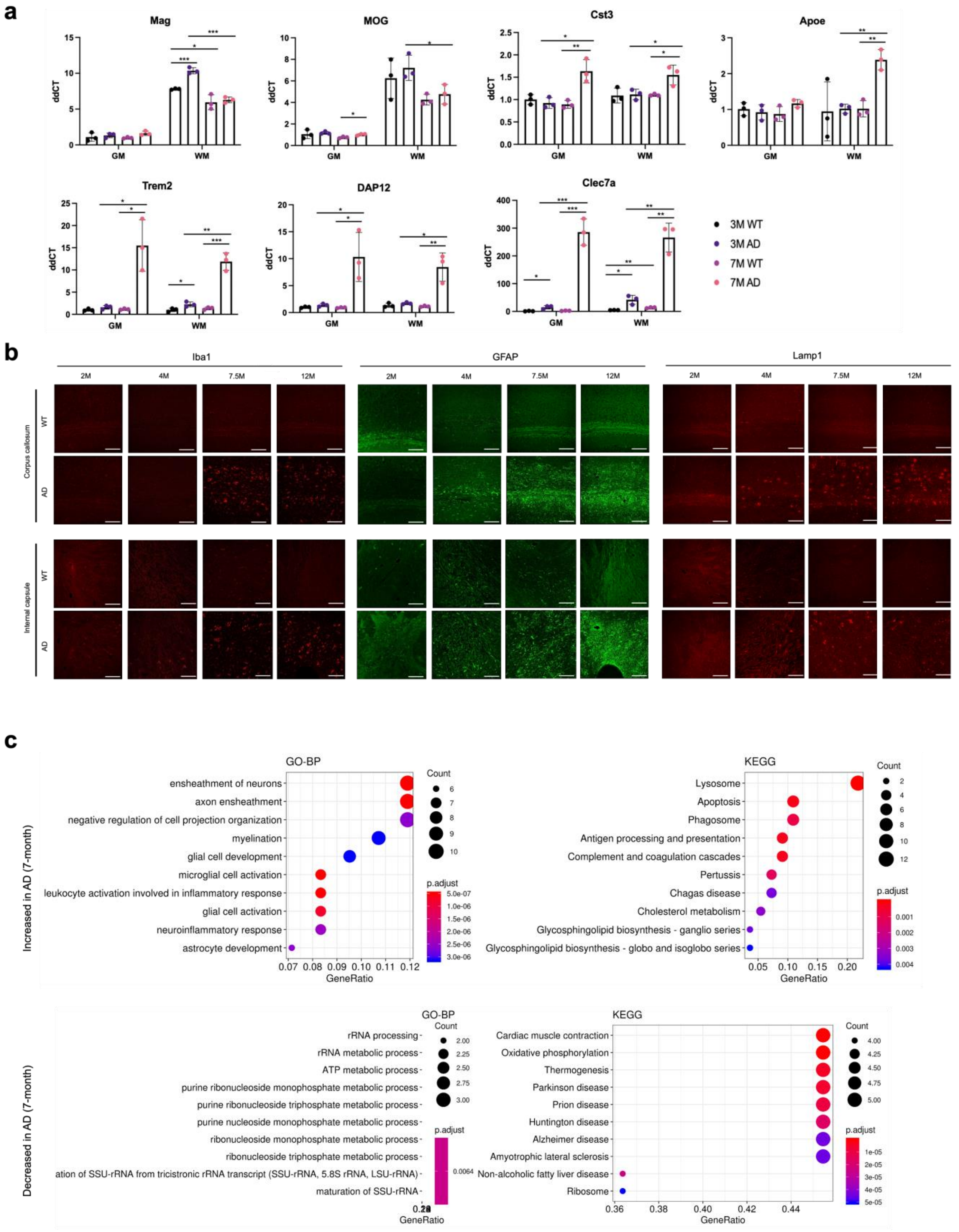

Supplementary Fig. 4. Changes of gene expression in white matter

(a) qPCR analysis of *Mag, MOG, Cst3, Apoe, Trem2, Tyrobp,* and *Clec7a* in the different groups of mice. Each symbol represents an individual mouse. *p<0.05, **p<0.01, ***p<0.001 (one-way ANOVA). Scale bars, 50 μm. (continue)

(b) Immunostaining of microglia/macrophages (Iba1, red), astrocytes (GFAP, green), and lysosomal function (Lamp1, red) were observed at the indicated ages (n=3). Notably, images showed increased reactive astrocytes, microglia, and lysosomal function in WM, internal capsule and corpus callosum, at 4-month AD model. Scale bars, 100 μm. (c) Functional term of differentially expressed genes of cluster 5 (white matter) in 7-month-old AD model. GO terms and KEGG pathways of upregulated genes in 7-month-old AD model were represented (above). In addition, those of downregulated genes in 7-month-old AD model were also represented (below).

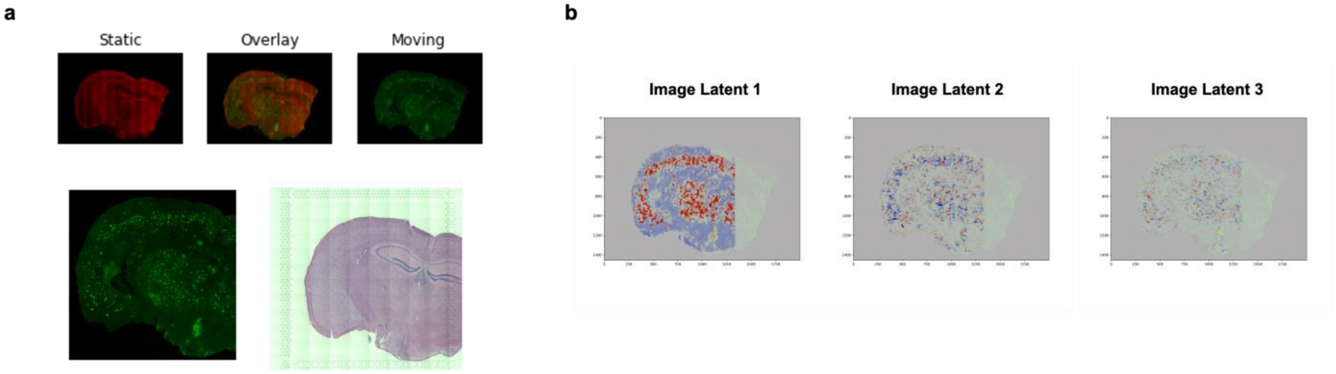

Supplementary Fig. 5. Registration of the IF image to H&E and image latent features.

(a) The IF image of 6E10 antibody represented Aβ accumulation acquired from 7-month-old AD model, a same animal with spatially resolved transcriptome. The IF image was coregistered with H&E image obtained for spatial transcriptomic data. (b) The spatial distribution of 2nd and 3rd image latents, derived from SPADE algorithm, was presented. These distribution patterns were visually different from Aβ accumulation.

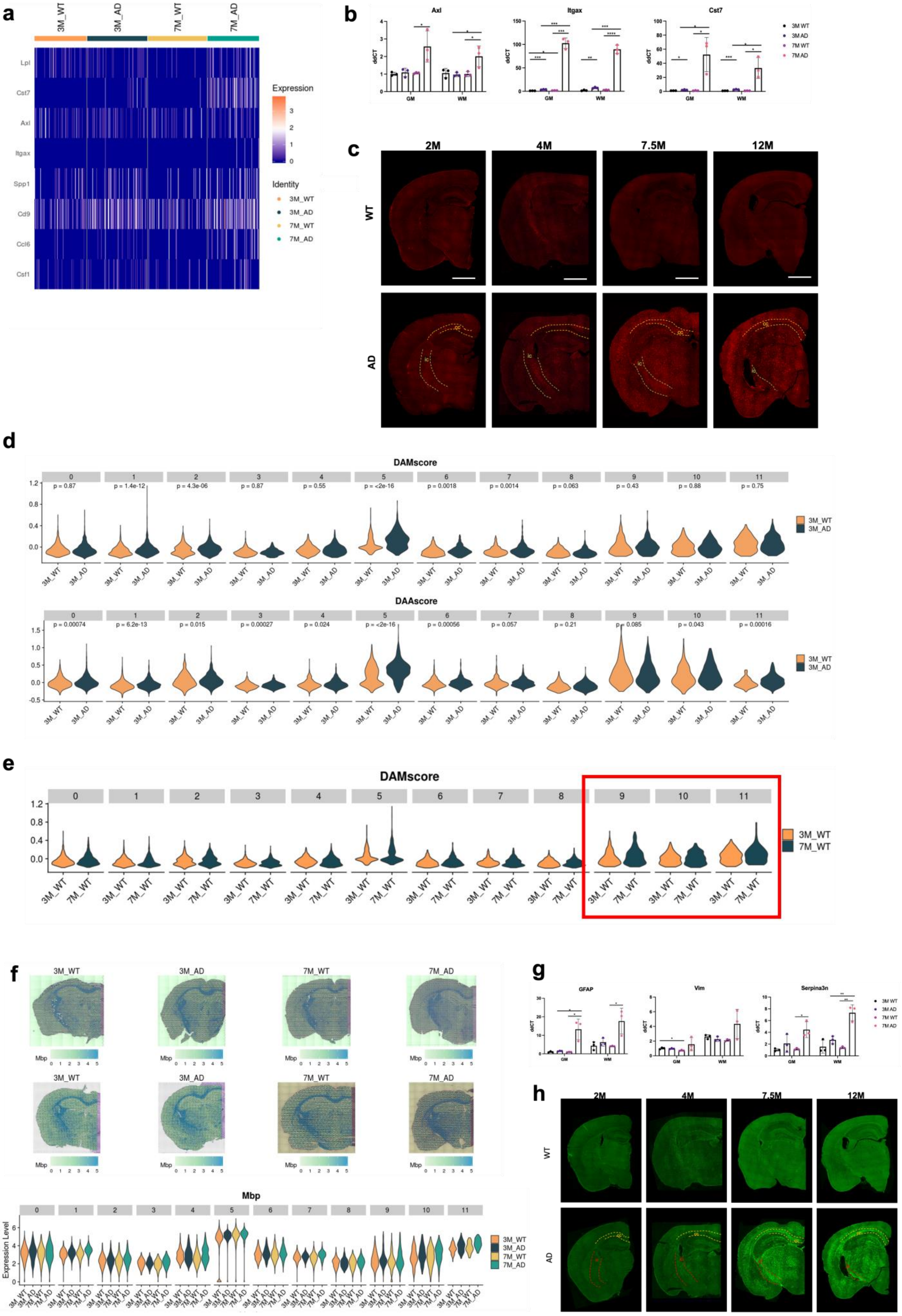
a
3M_WT
3M_AD
7M_WT
7M_AD
Lpl
Cst7
Axl
Itgax
Spp1
Cd9
Ccl6
Csf1
Expression
Identity
b
Axl
Itgax
Cst7
ddCT
GM
WM
3M WT
3M AD
7M WT
7M AD
c
2M
4M
7.5M
12M
WT
AD
cc
ic
d
DAMscore
p = 0.87
p = 1.4e-12
p = 4.3e-06
p = 0.87
p = 0.55
p = <2e-16
p = 0.0018
p = 0.0014
p = 0.063
p = 0.43
p = 0.88
p = 0.75
DAAscore
p = 0.00074
p = 6.2e-13
p = 0.015
p = 0.00027
p = 0.024
p = <2e-16
p = 0.00056
p = 0.057
p = 0.21
p = 0.085
p = 0.043
p = 0.00016
e
DAMscore
3M_WT
7M_WT
f
Mbp
Expression Level
g
GFAP
Vim
Serpina3n
h
2M
4M
7.5M
12M
WT
AD

Supplementary Fig. 6. Disease-associated microglia (DAM) signatures and astrocytes

(a) A heatmap for DAM signature genes according to mice. (b) qPCR analysis of *Axl, Itgax, and Cst7* in the different groups of mice. Each symbol represents an individual mouse. *p<0.05, **p<0.01, ***p<0.001 (one-way ANOVA). Scale bars, 1 mm. (c) IF with anti-Iba1 revealed increased microglia according to aging in the AD model (n=3). Notably, fluorescence signal was identified in internal capsule and corpus callosum in 4-month AD model, and the activity was clearly increased in cortex and thalamus at 7.5 months. (d) Statistical comparison of DAM and DAA scores of each cluster. Statistical tests were performed by Mann-Whitney test. (e) Three clusters of WT-mice showed relatively high DAM score compared with other clusters. These brain regions meninges (cluster 9), choroid plexus (cluster 10) and reticular nucleus of thalamus (cluster 11). (f) A marker of oligodendrocyte, Mbp, was not changed between groups while increased DAM or DAA signatures in WM. (g) qPCR analysis of *Gfap, Vim, and Serpina3n* in the different groups of mice. Each symbol represents an individual mouse. *p<0.05, **p<0.01, ***p<0.001 (one-way ANOVA). Scale bars, 1 mm. (h) IF with anti-GFAP revealed increased the activation of astrocytes according to aging in the AD model (n = 3). Notably, fluorescence signal was identified in internal capsule and corpus callosum in 4-month AD model, and the activity was clearly increased in cortex and thalamus at 7.5 months.

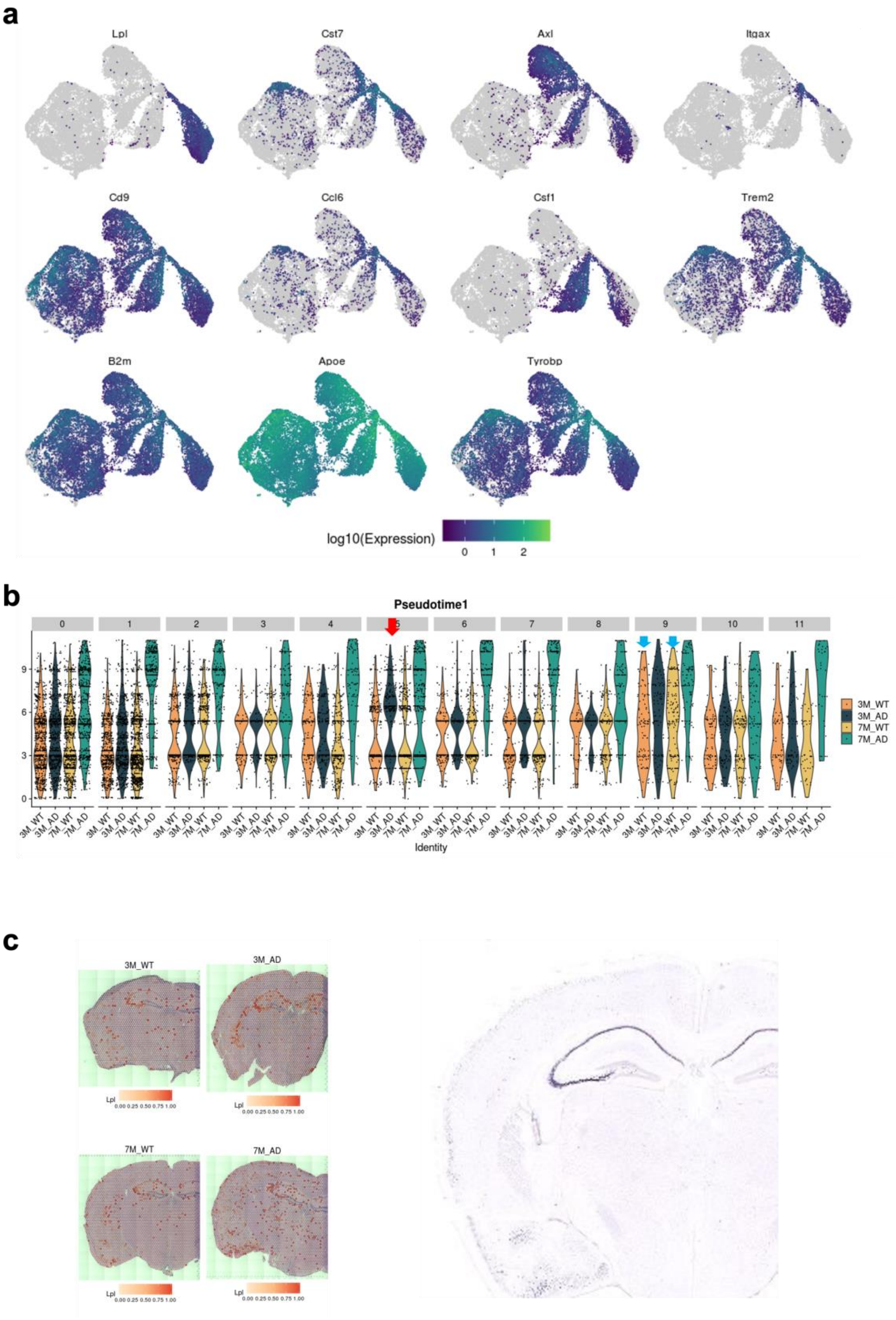

Supplementary Fig. 7. The expression of microglial genes and trajectory analyses

(a) The expression of key genes of microglia was represented by a color map with log10 (Expression) with UMAP plots drawn by microglial gene sets. (continue)

(b) The pseudotime of ‘trajectory 1’ of microglia according to mice. The pseudotime of trajectory 1 represented microglial activation in various clusters of the 7-month-old AD model. Notably, the relatively high pseudotime was found in cluster 5 (White matter) of 3-month-old AD model (Red Arrow). In addition, spots with relatively high pseudotime was found in WT. These spots were included in cluster 9 (meninges, subdural area) (Blue arrows). (c) The trajectory 3 was associated with AD-specific high pseudotime and characterized by high *Lpl*. *Lpl* expression was found in the hippocampus of WT as well as AD (*left*). This spatial pattern of *Lpl* expression was also identified by in situ hybridization data (ISH) from the Allen Brain Atlas (*right*).

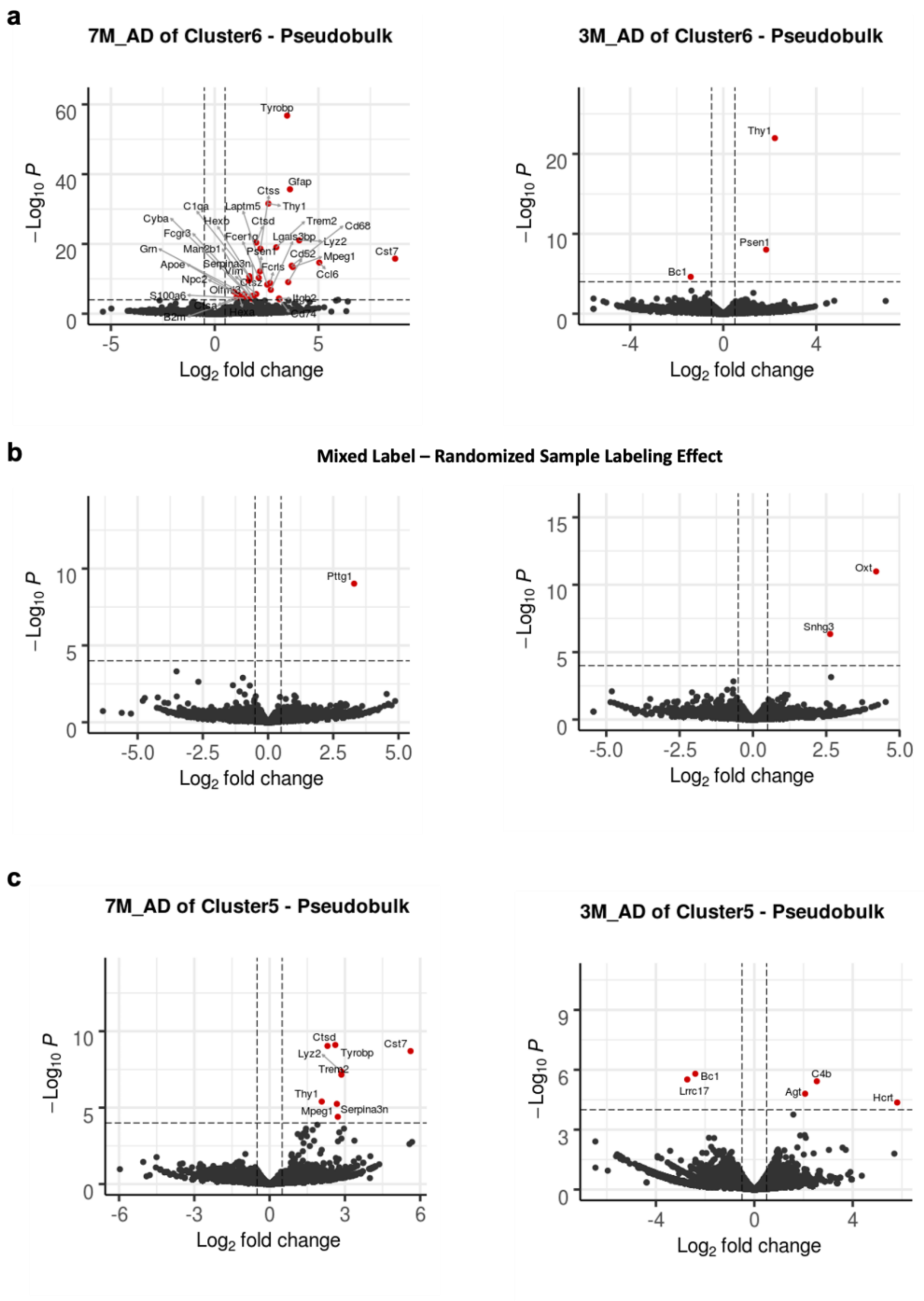

Supplementary Fig. 8. Comparison of AD model and control using pseudo-bulk RNA-seq. To ensure the limitation of analysis using spots of spatial transcriptomics accounting for individual biological variation and avoid potential biases, we attempted a pseudo-bulk RNA-seq by summing gene counts from each spot within a cluster and compared the groups. After summing gene counts of each cluster for each spatial transcriptomics sample, pseudo-bulk RNA-seq data for each brain region of 5XFAD and WT-mice were compared. The samples were annotated by grouping them and the DEG analysis was performed by randomly assigning the sample groups as well as original group to test how many genes were identified as DEGs. DESeq was used to perform the analysis on pseudo-bulk RNA-seq. (a) The comparison of 7-month-old and 3-month-old 5XFAD mice versus age-matched wild-type mice in cluster 6 was depicted. (b) The result of shuffling the group labels of the samples showed few number of DEGs compared with (a). (c) The comparison of 7-month-old and 3-month-old 5XFAD mice in cluster 5 using the same pseudo-bulk RNA-seq analysis.

Supplementary Tables

Supplementary Table 1. Primers for qPCR

**Oligonucleotides (qPCR)**	
Mag (Forward)	TAGGGTGAAAGACCCCCAGG
Mag (Reverse)	AGGGAACTGCAATGAGGAGC
MOG (Forward)	ATCTGGCAAGGGTGACGTGG
MOG (Reverse)	CAAGAAGCCCGAAGGGAGAA
Cst3 (Forward)	GCAGCTCGTGGCTGGAGTGAA
Cst3 (Reverse)	AGTGTGTGCCTTTCCAGG
Apoe (Forward)	ACAGATCAGCTCGAGTGGCAAA
Apoe (Reverse)	ATCTTGCGCAGGTGTGTGGAGA
Trem2 (Forward)	GGAACCGTCACCATCACTCT
Trem2 (Reverse)	ATGCTGGCTGCAAGAAACTT
Tyrobp (Forward)	GATTGCCCTGGCTGTGTACT
Tyrobp (Reverse)	CTGGTCTCTGACCCTGAAGC
Axl (Forward)	GGAGGAGCCTGAGGACAAAGC
Axl (Reverse)	TACAGCATCTTGAAGCCAGAGTAGG
Itgax (Forward)	CAAGACAGGACATCGCTCCC
Itgax (Reverse)	GTGAACAGTTGGTGACACTCT
Cst7 (Forward)	CCCTGGCTCCACAGTTTTGA
Cst7 (Reverse)	AGTCCAGCCCCCTGATGAC
Gfap (Forward)	GGCGCTCAATGCTGGCTTCA
Gfap (Reverse)	TCTGCCTCCAGCCTCAGGTT
Vim (Forward)	AAGAGATGGCTCGTCACCTT
Vim (Reverse)	GGGTGTCAACCAGAGGAAGT
Serpina3n (Forward)	TCTGTAGGTAAAGCCCAGGAT
Serpina3n (Reverse)	TGATGGAGGCCAATGTGAGAC
beta-actin (Forward)	AAGACCTCTATGCCAACACAGT
beta-actin (Reverse)	GCTCAGTAACAGTCCGCCTA

Supplementary Table 2. Anatomical regions of clusters

Cluster	Anatomical Structure	Top Upregulated Genes				
0	Hypothalamus	Oxt	Resp18	Nap1l5	Sparc	Trh
1	Thalamus	Prkcd	Tcf7l2	Pcp4	Ntng1	Slc17a6
2	Hippocampus	Hpca	Wipf3	Ddn	Gria1	Ptk2b
3	Cortex (Outer layers)	Nrgn	Lamp5	Camk2n1	Mef2c	Egr1
4	Caudate Putamen	Penk	Gpr88	Tac1	Ppp1r1b	Pde10a
5	White matter	Plp1	Cryab	Mobp	Cldn11	Mal
6	Cortex (Inner layers)	Vxn	Ttc9b	Rprm	Nr4a2	Tbr1
7	Cortex (Retrosplenia, layer 5)	Lamp5	Thy1	1110008P14Rik	Dkk3	Nrgn
8	Pyriform/Amygdala	Nptxr	Olfm1	Lmo3	Slc30a3	Arpp21
9	Meninges	Ptgds	Vtn	Igf2	Mgp	Acta2
10	Choroid Plexus	Gm5741	Gng8	Ttr	Ecrg4	Enpp2
11	Reticular nucleus of thalamus	Pvalb	Gad1	Six3	Gad2	Pcp4l1

Supplementary Table 3. Differentially expressed genes of AD mouse brain versus wild-type (*Separated Excel File*)

Supplementary Table 4. List of curated gene sets for evaluating spatiotemporal microglial patterns

**Microglial Gene Sets**
Hexb
Cst3
Cx3cr1
Ctsd
Csf1r
Ctss
Sparc
Tmsb4x
P2ry12
C1qa
C1qb
Tmem119
Tyrobp
Ctsb
Apoe
B2m
Fth1
Lyz2
Trem2
Axl
Cst7
Ctsl
Lpl
Cd9
Csf1r
Ccl6
Itgax
Timp2
